# Molecular characteristics and regulatory effects of dwarfing *Rht* genes in *Triticum aestivum* L

**DOI:** 10.1186/s12870-025-07556-w

**Published:** 2025-11-10

**Authors:** Ying run Wang, Jia Shi, Yi yang He, Yu lu Tao, Lan tian Ren, Qing qin Shao, Xiang Chen, Zi feng Wu, Jin cai Li, Jia cheng Zheng

**Affiliations:** 1 College of Agronomy, Anhui Science and Technology University, Fengyang, Anhui China; 2 Anhui Engineering Research Center for Smart Crop Planting and Processing Technology, Fengyang, Anhui China; 3 National Engineering Laboratory of Crop Stress Resistance Breeding, Anhui Agricultural University, Hefei, Anhui China; 4 Anhui Agricultural Technology Station, Hefei, Anhui China

**Keywords:** Wheat, *Rht* dwarfing genes, Functional characteristics, Expression patterns, Regulatory effects

## Abstract

**Background:**

Application of *Rht* dwarfing genes has significantly enhanced plant lodging resistance and harvest index. However, the numerous members of *Rht* genes, particularly newly cloned genes in recent years, remain poorly understood in integrated analysis of their expression patterns under stress conditions, allelic interaction effects, and molecular regulatory mechanisms, these limit our comprehensive exploration of *Rht* gene regulatory network, and hinder the potential application in wheat genetic improvement.

**Results:**

This study comprehensively analyzed the characteristics of *Rht* family members. The results showed that there were 27 *Rht* genes in wheat, with only 10 cloned members. Collinearity of *Rht* genes was observed in wheat genomes, as well as cross-species collinearity. *Rht1* exhibited significantly highest basal expression from roots to grain, and actively responded to phosphate stress, with markedly upregulated after 4 h treatment, achieving a maximum value of log_2_ fold change = 4.02. The interaction network was centered around Rht4, Rht12, and Rht24 proteins. In 47 wheat varieties, *Rht24* was more widely distributed, *Rht1* + *Rht2* exhibited a strong dwarfing effect, whereas showing negative impact on thousand kernel weight (TKW), and introduction of *Rht12* or *Rht24* can mitigate the negative effect. A stack of *Rht4* + *Rht8* can optimizes plant architecture, while the combination of *Rht8*, *Rht12* and *Rht24* achieves higher yields, ranging from 9150 kg·ha⁻¹ to 11,250 kg·ha⁻¹, such as cultivars SN25, SN29 and XN579. The distribution frequency of *Rht5* was correlated with plant height and yield-related traits, with all correlation coefficients (r) exceeding 0.89 and false discovery rate (FDR) being less than 0.05. *Rht8* and *Rht12* exhibited strong correlations with plant height and TKW, respectively, with r value all exceeding 0.8.

**Conclusions:**

This study systematically analyzed the regulatory relationships among *Rht* genes and their expression patterns under abiotic stress conditions, and evaluated the combined effects of alleles. Grey correlation analysis revealed that *Rht1* and *Rht5* were strongly correlated with plant height and yield-related traits. These provide theoretical support for allele combinations to breed the dwarf and high-yield wheat during molecular design breeding, such as *Rht4* + *Rht8* for reasonable plant type, *Rht8* + *Rht12* + *Rht24* for favourable grain yield, thereby achieving breeding goals that balance height and stable yields.

**Supplementary Information:**

The online version contains supplementary material available at 10.1186/s12870-025-07556-w.

## Introduction

The dwarf genes (*Rhts*) are a key factor in wheat dwarf breeding. The functional deficiency or reduced expression of *Rht* genes leads to plant dwarfism [1]. Dwarfism not only enhances wheat resistance to lodging but also improves its tolerance to high water and fertilizer conditions, and increases the harvest index. In the 1960 s, the dwarf genes *Rht-B1b* and *Rht-D1b* were introduced into wheat varieties, triggering the first “green revolution”. Currently, over 70% of commercial wheat varieties contain *Rht-B1b* and *Rht-D1b* genes, significantly reducing plant height by 20%, and enhancing lodging resistance, with average yield increase ranging from 5% to 25%. However, these genes were accompanied by drawbacks, such as shortened coleoptile length and reduced TWK [2]. Currently, through QTL mapping, linkage map construction, and molecular markers, 27 *Rht* genes have been identified [3]. However, a few *Rht* genes were cloned, and even fewer superior dwarf genes were available for utilization, the regulatory mechanisms of many *Rht* genes remain unclear. Therefore, it is necessary to identify and utilize dwarf genes during the development of high-yielding, stress-tolerant, and adaptable dwarf wheat varieties.

Gibberellic acid (GA) is an important plant hormone that regulates plant height. Based on the sensitivity of wheat dwarf mutants to gibberellic acid, *Rht* is classified into GA-sensitive and GA-insensitive types [4]. The *Rht-B1b* and *Rht-D1b* genes are GA-insensitive dwarfing genes that encode DELLA proteins in the GA signaling pathway. Through translational reinitiation, they produce N-terminally truncated proteins due to premature termination of amino acid translation, leading to plant dwarfism [5]. GA-sensitive genes can be classified into two types: one type encodes gibberellin oxidases that directly participate in GA metabolism, such as the *Rht12* (*TaGA2oxA13*) and *Rht18* (*GA2oxA9*), which influence stem elongation by regulating active GA levels. The other type modulates GA signaling through non-oxidase regulators, such as the *Rht8* (RNHL + ZF-BED), *Rht25* (PLATZ) and *Rht26* (PMEI). *Rht8* gene encodes an RNase H-like protein containing a ZF-BED domain. This protein reduces the levels of active gibberellin GA_3_ while increasing GA_4_ content through regulating the expression of *GA3ox* and *GA20ox-2*, resulting in plant dwarfism [6]. However, many individual dwarf genes have negative effect on wheat, such as reduced grain number, TWK, and protein content, combining dwarf genes compensate for the negative effect. The combination of *Rht1* + *Rht15* significantly increases grain yield and resistance to lodging [7], *Rht2* + *Rht13* significantly reduces plant height without compromising yield, while compensating for the negative effect of *Rht13* on flag leaf width and *Rht2* on coleoptile length [8], *Rht4* + *Rht8* compensates for the negative effect of *Rht4*, *Rht8* + *Rht13* has no additive effect on plant height and yield, and *Rht13* has a delaying effect on flowering time [9].

This study analyzed the genomic structural features of 10 cloned *Rht* genes in wheat, including conserved motifs, domain composition, *cis*-acting elements of promoters, intraspecific and cross-species collinearity, and gene regulatory networks, to identify their conserved functional modules. Simultaneously, under treatments of various abiotic stress, such as drought, salt stress, GA, phosphorus, low and high nitrogen, ABA and SA, the expression profiles of different *Rht* genes were analyzed to determine response mechanisms. Furthermore, the molecular markers closely linked with *Rht* genes were used to detect the allelic variation in 47 major wheat cultivars from the Huang-Huai-Hai region, to determine the distribution frequency of each *Rht* gene. Through integrating data between distribution frequency and plant height, yield-related traits, the genotype-phenotype associations were analyzed, to evaluate the polygenic effects of *Rht* genes. The comprehensive stress response analysis of cloned *Rht* genes relatively remains scarce, hindering systematic elucidation of their molecular characteristics on plant height and yield-related trade-offs by preponderant allele combination. This study is to fill this gap and provides theoretical basis for parent selection, gene polymerization and stress-resistance breeding in developing high-yield and dwarf wheat varieties.

## Materials and methods

### Field experiments and planting design

47 major wheat cultivars were collected from the Huang-Huai-Hai region. The genetic backgrounds of 47 cultivars are representative, with a broad genetic nature population, due to their relatively independent breeding pedigrees and stronger competent adability within the major crop region. The field trials were consecutively conducted at both crop seasons of 2022–2023 and 2023–2024 in the experimental fields of Anhui Science and Technology University. The trials employed a Randomized Complete Block Design (RCBD) with three replications. Each plot comprised 32 rows, 1.5-meter row long with 0.25-meter row spacing, and a seeding rate of 225 kg/ha. All plots received uniform conventional agricultural management practices, including irrigation, fertilization, and weed control.

### Sample collection and DNA extraction

At the wheat three-leaf stage (Z12) [10], a single plant of each variety with consistent growth was selected from each plot, to collect leaf samples, and then rapidly freeze in liquid nitrogen, grind and extract genomic DNA by using the CTAB method [11].

### Agronomic trait survey

At the mid-grain-filling stage (Z73). The main-stem plants, free of pests and diseases, were randomly selected from each plot, plant height from stem base to spike apex was measured. At harvest (Z93), main stem spikes length (MSSL) was randomly determined from the rachis base to tip, without awns. Main stem spikes were manually threshed to determine the main stem spike grain numbe (MSSGN) and thousand kernel weight (TKW). After, the grain yield (GY) was estimated by threshing weight per unit area (6m^2^). Three replicates were conducted for the GY, as well as ten replicates in each plot for the other traits.

### RNA and cDNA sample Preparation

Wheat variety Chinese Spring was used to cultivate and treat as described by Zheng et al. [12]. Adverse stress included PEG_6000_ (20%, W/V), NaCl (150 mmol/L), gibberellin (GA_3_, 200 µmol/L), phosphorus solution (10 mg/L), low nitrogen (ammonium nitrate, 0.5 mmol/L), high nitrogen (2.5 mmol/L), abscisic acid (ABA, 1000 µmol/L), and salicylic acid (SA, 0.6 mmol/L). The control was water culture. Treatment durations included 0.5 h, 1 h, 2 h, 4 h, 6 h, 12 h, 24 h, and 48 h, with three biological replicates. After treatment, roots, stems, and leaves were mixed to rapidly freeze in liquid nitrogen for RNA extraction. Total RNA was extracted using Trizol (TaKaRa, T9108, Japan). cDNA was synthesized using a Reverse Transcription Kit (Beyotime, D7178M, China) and stored at −80℃ for later use. Experimental procedures followed the reagent instructions.

### *Rht* gene analysis in wheat

#### Sequence organization of *Rht *genes

Through literature review, 27 members of *Rht* genes were identified with basic information on gibberellin (GA) response, genetic background, chromosomal localization, and linkage markers (Table 1). Gene numbers of 10 cloned *Rht* genes were used to blast in the NCBI database, to obtain the corresponding sequences of each *Rht* gene, and confirm in the Ensembl Plants database (Table 2). The Conserved Domain database of NCBI, the Pfam database and SMART database were used to perform the conserved domain structure of Rht proteins, and remove redundant residues. The URL information of database was listed in Appendix Table S1, the same was as below.

#### Physicochemical properties and subcellular localization of *Rht* members

The Protein Parameter Calc module of TBtools software was used to analyze the molecular weight and physicochemical properties of 10 *Rht* members, and the WOLF PSORT database was used to predict the subcellular localization characteristics.

#### *Rht* member alignment and phylogenetic tree construction

The amino acid sequences of 10 Rht proteins were aligned by the MAGEx64 software, imported into GeneDoc software for visualization, and characterized the features of Rht amino acids. The aligned amino acid sequences were used to construct a phylogenetic tree with the neighbor-joining method (a bootstrap threshold of 1000), and visualization modification by the Chiplot online tool. The information of Rht proteins was shown in Appendix Table S2.

#### Conserved motifs, gene structure, and promoter elements of *Rht* members

The genome sequence, amino acid sequence, and annotation files (IWGSC.58 version) of wheat variety Chinese Spring were downloaded from the Ensembl Plants database, to retrieve the locations of exon, intron, and non-coding region for *Rht* genes. The Motifs and functional domains of Rht proteins were analyzed by using the MEME database, the online-generated files were imported into TBtools software for visualization. The promoter elements were predicted in the PlantCARE database [41]. Detailed information on the Motifs and promoter elements were listed in Appendix Table S3 and Table S4, respectively.

#### Collinearity analysis of *Rht* genes

The genome data and annotation information of *Brachypodium distachyon* L. (Brachypodium_distachyon_v3.0), *Hordeum vulgare* L. (Hordeum_vulgare.MorexV3) and *Zea mays* L. (Zea_mays.Zm-B73-Reference-nam-5.0) were download from the Ensembl Plants database. The colinearity relationships of *Rht* genes within wheat, and between wheat and *Brachypodium distachyon*, barley, or maize were analyzed by the TBtools software, and created the colinearity diagram based on the Advanced Circos module. The anchors, block size and Ks values of *Rht* homologous gene pairs between wheat and *Brachypodium distachyon*, barley, maize, were calculated by using the Simple Ka/Ks Calculator Module in TBtools software, as well as *Rht* gene pairs within wheat segmental duplications, The colinearity data was shown in Appendix Table S5.

#### Interaction and GO enrichment of *Rht* members

The interaction network of Rht protein was predicted by the STRING database, and visualized with Cytoscape_v3.9.1 software. The GO enrichment analysis of biological process, cellular component, and molecular function for Rht members was predicted by the online Shiny GO tool, with a false discovery rate (FDR) at *P <* 0.01 level. The relevant data was listed in Appendix Table S6.

#### miRNA analysis targeting *Rht* genes

Based on the sequence of *Rht* genes, the target sites of *Rht* genes were predicted to select corresponding miRNA molecules in the psRNATarget database, and the interaction network between *Rht* genes and corresponding miRNA was constructed by using the Cytoscape_v3.9.1 software. The data was listed in Appendix Table S7.

#### Expression analysis of *Rht* genes

##### Tissue-specific expression

The transcriptomic data (FPKM values) was downloaded from the Wheatomics database for the wheat Chinese Spring tissues of roots, stems, leaves, spikes, and grains, organized for each *Rht* gene, and plot a heat map by TBtools software.

##### Expression patterns under different adverse stress and hormone induction

*TaActin* (GenBank: AB181991.1 and LOC125519869) was used as an internal reference gene to standardize the expression of the target gene. The expression level of *Rht* genes was detected based on wheat cDNA samples under different treatments, using a Real-time quantitative PCR instrument (Thermo Fisher Scientific, ABI7500). The real-time quantitative PCR (RT-qPCR) reaction procedures and system were according to Dai methods [42]. The RT-qPCR primers were listed in Appendix Table S8.

##### Detection of *Rht* genes in different wheat varieties

The closely linked molecular markers of 10 cloned *Rht* genes were selected, and only eight genes were obtained. The primers were verified in the Grain genes database, and then to test the 47 wheat varieties. Through establishment of negative control sample (Chinese Spring variety without any *Rht* genes), positive control variety Burtert937 with *Rht4*, Marfed M with *Rht5*, JM23 with *Rht8*, AK58 with *Rht12*, Chris Mutant with *Rht17*, SX828 with *Rht24* gene, were used to verify the target accuracy of 47 wheat varieties for the *Rht* genotyping results [9, 16, 22, 30, 36, 43–49], the seven varieties were provided in our lab (Appendix Table S9). The PCR system and reaction procedure were referred to Meng [50]. The molecular marker results were labeled as “+” for target gene bands, otherwise with labele as “-” signal. The data results were listed in Appendix Table S10.

## Data processing and analysis

Relative quantitative analysis of RT-qPCR data was performed with log_2_ fold change values (log_2_ FC) by using the 2^−ΔΔCt^ method [51], and data was organized and visualized using TBtools software. Statistical analysis was performed using one-way analysis of variance (ANOVA) combined with Tukey’s test. The p-values were corrected by using the Benjamini-Hochberg method, to control the FDR. All data analyses were conducted using GraphPad Prism 10.1.2 software. The agronomic traits of 47 wheat cultivars were concluded by Origin 2021 software, and processed cluster analysis by SPSS 18.0 software. To evaluate the effects of year, variety, and year×variety, a combined analysis of variance (ANOVA) over two-year period was performed using SPSS 18.0 software. For 47 wheat varieties, the detected allele fragment size with the eight pairs of selected marker was statistically analyzed, converted into a genetic similarity matrix by the NTsys2.10e software, and constructed a cluster analysis by using UPGMA method. The allele distribution results were converted into a binary 0,1 matrix, and gray association analysis was performed between allele distribution and agronomic traits by using Excel 2010 software [52].

## Results and analysis

### Identification of wheat *Rht* members

A total of 27 *Rht* dwarf genes are identified, *Rht* genes are distributed in diploid, tetraploid, and hexaploid wheat (Table 1). Except for *Rht1*, *Rht2*, *Rht3*, *Rht21*, and the alleles of *Rht1* (*Rht11* and *Rht17*) and *Rht2* (*Rht10*), the remaining dwarf genes exhibit sensitivity to GA. *Rht1*, *Rht2*, and *Rht17* are semi-recessive genes, while *Rht4*, *Rht6*, *Rht7*, *Rht8*, *Rht9*, *Rht11*, *Rht13*, *Rht20*, and *Rht22* are recessive genes, *Rht3*, *Rht12*, *Rht14*, *Rht15*, and *Rht18* are dominant genes, and *Rht5*, *Rht10*, *Rht16*, *Rht19*, *Rht21*, and *Rht23* are semi-dominant genes. In chromosome distribution, *Rht1*, *Rht2*, *Rht6*, and their alleles are distributed on chromosome 4, *Rht7* and *Rht21* are located on chromosome 2 A, with *Rht9* and *Rht12* on chromosome 5 A, and *Rht14*, *Rht16*, *Rht18*, and *Rht25* are distributed on chromosome 6 A, whereas *Rht15*, *Rht19*, and *Rht20* have not been clearly identified.

For the the stem-shortening effect, *Rht4* mutation reduced TKW and GY by 12.5%, and 30%, respectively, *Rht15* mutation resulted in a decrease of 12.9% and 10.3% in TKW and GY, respectively, while *Rht25* showed weak effect on TKW and GY, with a decrease of 4.1% and 5.5%, respectively [9, 28, 38]. In contrast, *Rht2* mutation can increase the GY by 25.7%, whereas it weakens the resistance to Fusarium head blight (FHB) [53]. For other *Rht* genes, *Rht18* mutation enhances both yield and grain number, while *Rht1* and *Rht9* mutations increase GY but decrease TKW. Similarly, *Rht13* mutation raises grain number yet reduces TKW, *Rht7* mutation reduces GY, *Rht14* mutation decreases TKW, *Rht22* mutation increases grain number, and *Rht24* mutation increases TKW.

**Table 1:** Member information and functional characteristics of *Rht* genes of wheat

Rht genes	Gene mutants	Gene symbol	GA response	Recessive/dominant genes	Genetic background	Position (chromosome)	Markers	Traits	References
*Rht1*	*Rht-B1b*	KC767925.1	GAI	Semi-recessive	Norin 10×Brevor 14	4BS	BF/MR1	20%PH; Y/I; TKW/R; GN/I	[2, 13]
*Rht2*	*Rht-D1b*	KC767927.1	GAI	Semi-recessive	Norin 10×Brevor 14	4DS	DF/MR2	20%PH; Y/I; FHB/S	[2, 14]
*Rht3*	*Rht-B1c*	JF930279.1	GAI	Dominant	Minister dwarf	4BS	Xpsr584/Xpsr144	64%PH	[14, 15]
*Rht4*	*Undefine*		GAR	Recessive gene	Burt ert 937	2BL	Xwmc317	12%PH; Y/R; TKW/R	[9]
*Rht5*	*Undefine*	LOC123064363	GAR	Semi-dominant	Marfed Mutant	3BS	Xbarc102	50%PH	[16, 17]
*Rht6*	*Undefine*		GAR	Recessive gene	Burt Mutant	4D		PH	[18]
*Rht7*	*Undefine*		GAR	Recessive gene	Bersee Mutant	2AS		24%PH; Y/R	[19]
*Rht8*	*Undefine*	LOC123172274	GAR	Recessive gene	Akakomugi	2DS	Xgwm261/Xwmc503	20%PH	[6]
*Rht9*	*Undefine*		GAR	Recessive gene	Akakomugi	5AL	BARC151	25%PH; Y/I; TWK/R	[20]
*Rht10*	*Rht-D1c*		GAI	Semi-dominant	Aibian	4DS	Xgwm634/Xpsr921	>64%PH	[21]
*Rht11*	*Rht-B1e*		GAI	Recessive gene	Karlik	4BS	Xgwm495/Xwmc48	PH	[21]
*Rht12*	*Rht12b*	KAF7062674.1	GAR	Dominant	Karcagi 522M7K	5AL	Xgwm291/Wmc410	46%PH; Y/I	[22, 23]
*Rht13*	*Undefine*		GAR	Recessive gene	Magnif Mutant	7BS	Gwm577/Wmc276	24%PH; GN/I; TKW/R	[24, 25]
*Rht14*	*Undefine*		GAR	Dominant	Castelporizano	6 A	Wmc753	26%PH; TKW/R	[26, 27]
*Rht15*	*Undefine*		GAR	Dominant	Durox	unknown		31.5%PH; Y/R; TKW/R	[28]
*Rht16*	*Undefine*		GAR	Semi-dominant	Edmore Mutant	6AS	Xbarc3	PH	[29]
*Rht17*	*Rht-B1p*		GAI	Semi-recessive	Chris Mutant	4BS		30%PH	[30]
*Rht18*	*Undefine*		GAR	Dominant	Icaro	6 A	IWA3230/IWB62878	24%PH; Y/I; GN/I	[31, 32]
*Rht19*	*Undefine*		GAR	Semi-dominant	Vic Mutant	unknown		PH	[33]
*Rht20*	*Undefine*		GAR	Recessive gene	Burt Mutant 860	unknown		PH	[33]
*Rht21*	*Undefine*		GAI	Semi-dominant	XN0004	2AS		PH	[34]
*Rht22*	*Undefine*		GAR	Recessive gene	Aiganfanmai	7AS	Xbag295.s53/Xb295.191	PH; GN/I	[1]
*Rht23*	*Undefine*		unknown	Semi-dominant	Nauh164	5DL	Xgdm63/Barc110	PH	[35]
*Rht24*	*Rht24b*	LOC123137545	GAR	unknown	Aikang 58	6DL	Xbarc103/Xwmc256	10%PH; TKW/I	[36, 37]
*Rht25*	*Rht25b*	KAF7078549.1	GAR	unknown	UC1110/PI610750	6AS		13%PH; Y/R; TKW/R	[38, 39]
*Rht26*	*Undefine*	KAF7039193.1	GAR	unknown	ZM175×LX987	3DL	KASP517/KASP518	PH	[40]
*Rht27*	*Undefine*		GAR	unknown	G1812	3 A	3T-387/3T-306	70%PH	[3]

*GAI* is gibberellin insensitive, *GAR* is gibberellin sensitive, *PH* is plant height, *Y* is yield, *TKW* is thousand kernel weight, *GN* is grain number for a spike, *I* is increasing effect, *R* is decreasing effect, *FHB* is Fusarium head blight, and *S* is susceptible

### Physicochemical properties and subcellular localization

For the 27 identified *Rht* members, only 10 genes have been cloned (Table 2). Among these, *Rht3*, *Rht11* and *Rht17* are alleles of *Rht1*. *Rht4*, *Rht5*, and *Rht26* have multiple candidate genes, and their major-effect genes have not been identified. The CDS (coding sequence) lengths of 10 *Rht* genes range from 732 to 2427 bp, encoding amino acids of 243–808 aa, with molecular weights ranging from 26.34 to 86.94 kDa, isoelectric points from 4.99 to 9.1, and aliphatic indices (AI) between 73.33 and 92.28. The instability index (INI) of Rht5 and Rht12 is below 40, indicating relatively stable proteins, while the other Rht proteins are predicted to be unstable. The grand average of hydropathy (GRAVY) is negative in ten members, indicating hydrophilic traits. Subcellular localization indicates that Rht4, Rht12, and Rht25 are localized in the cytoplasm, as well as Rht5 and Rht26 in the vacuole and chloroplast, respectively, the remaining Rhts are localized in the nucleus. 

**Table 2:** Physicochemical properties of *Rht* genes

Gene name	Gene ID	Amino Acid (aa)	CDS length (bp)	MW (kDa)	pI	INI	AI	GRAVY	Location
*Rht1(Rht11/Rht17)*	*TraesCS4B02G043100*	621	1866	65.17	4.99	50.05	78.82	−0.107	nucl
*Rht2*	*TraesCS4D02G040400*	623	1872	65.34	4.99	49.75	78.89	−0.125	nucl
*Rht3*	*Undefine*	651	1956	68.18	5.04	48.66	79.86	−0.108	nucl
*Rht4*	*TraesCS2B02G570900*	375	1128	41.34	5.49	39.61	75.47	−0.309	cyto
*Rht5*	*TraesCS3B02G025600*	523	1572	59.90	8.8	31.69	92.28	−0.144	vacu
*Rht8*	*TraesCSU02G024900*	808	2427	86.94	8.37	51.94	78.89	−0.235	nucl
*Rht12*	*TraesCS5A02G543100*	357	1074	38.72	5.69	30.7	79.52	−0.153	cyto
*Rht24*	*TraesCS6A02G221900*	347	1044	37.69	5.83	55.89	74.58	−0.171	nucl
*Rht25*	*TraesCS6A02G156600*	243	732	26.34	8.94	52.26	73.33	−0.345	cyto
*Rht26*	*TraesCS3D02G404000*	604	1815	64.72	9.1	42.49	82.91	−0.139	chlo

*MW* is molecular weight, *pI* is isoelectric point, *INI* is instability index, *AI* is aliphatic index, *GRAVY* is grand average of hydropathy, Location is subcellular localization, *nucl* is nucleus localization, *cyto* is cytoplasm, *vacu* is vesicle, and *chlo* is chloroplast

### Systematic evolutionary of *Rht* proteins

**Fig. 1:**
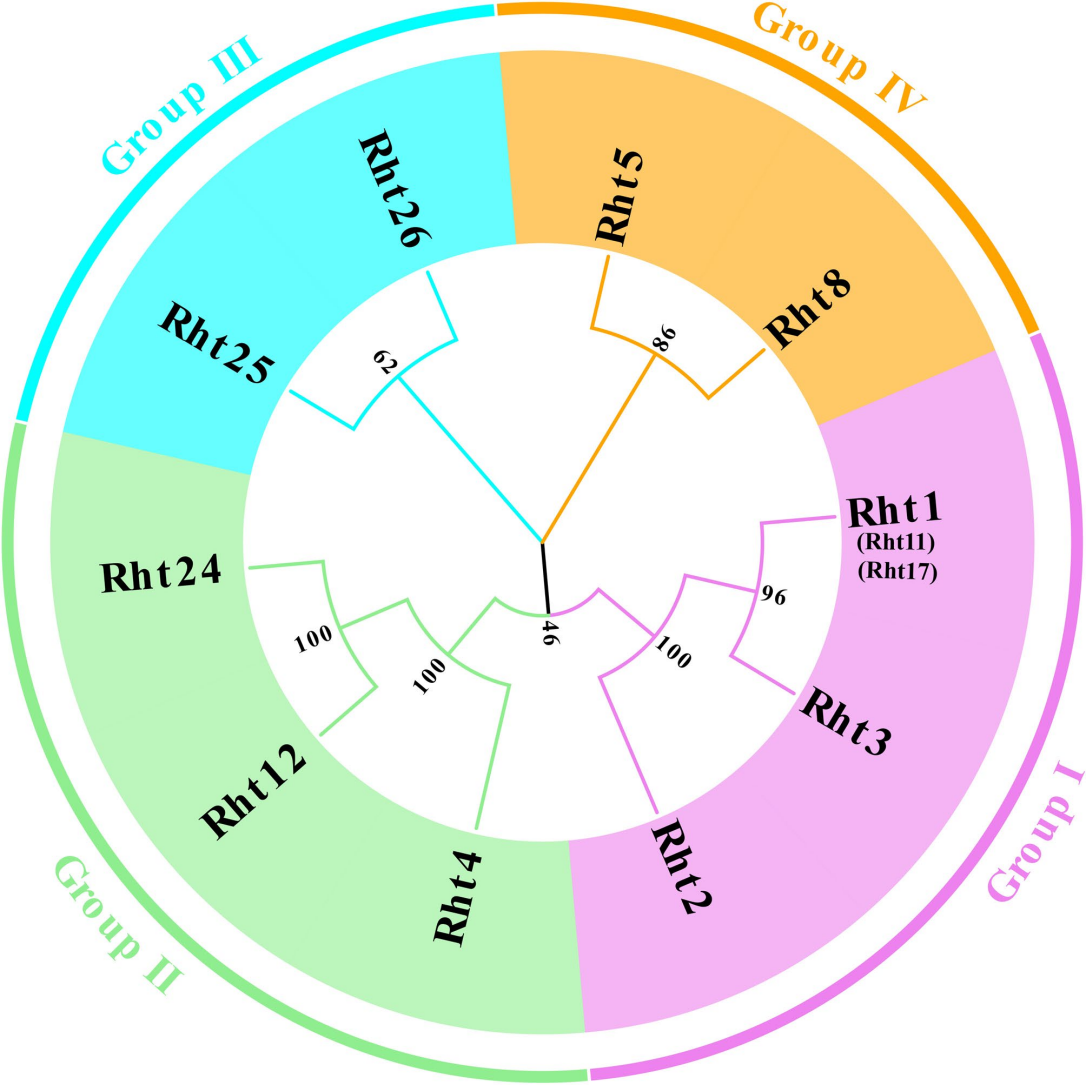
Phylogenetic relationships of cloned *Rht* members in wheat Note: The phylogenetic tree was constructed by method of neighbor-joining, with a bootstrap threshold of 1000. The numbers in the Figure represented the phylogenetic distance among different Rht proteins. Detailed information of Rht members was listed in Table S2

Ten Rht proteins are classified into four subgroups (Fig. 1). Group I includes three members, Rht1 (Rht11 and Rht17), Rht2, and Rht3, all belonging to DELLA family. Group Ⅱ includes three members, Rht4, Rht12, and Rht24, all belonging to the gibberellin oxidase family, which regulate plant height of wheat through the GA signaling pathway, and share similar functions in GA degradation metabolism. Although both Rht25 and Rht26 belong to Group Ⅲ, Rht25 is PLATZ family, regulating the gene transcriptional activity related to cell proliferation, while Rht26 is PMEI family, inhibiting pectin methylesterase activity and regulating cell elongation. Group Ⅳ includes Rht5 and Rht8, belonging to P450 family and Ribonuclease H (RNHL) superfamily, respectively. Mutations in both *Rht5* and *Rht8* have negative effect on GA biosynthesis and signaling pathways [17, 48].

### Gene structure, functional domains, and conservation characteristics

**Fig. 2:**
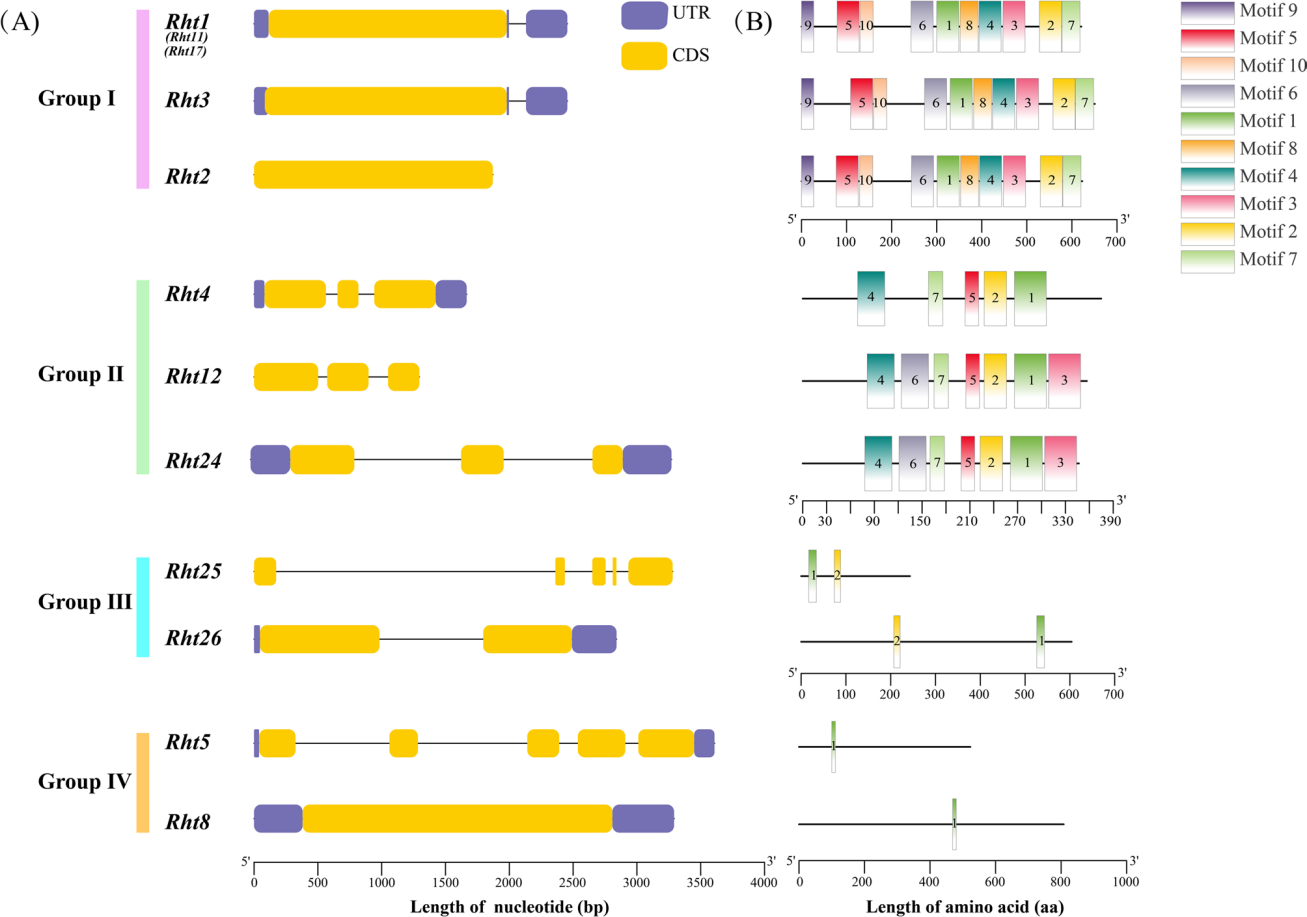
Gene structure and protein motif of 10 Rht members Note: **A** is gene structure of *Rht* members. **B** is the conserved motif of Rht proteins. Detailed information of *Rht* members was listed in Table S3

As shown in Fig. 2A, the exon number of *Rht* members ranges from 1 to 5. *Rht25* and *Rht5* have the most exon with 5 numbers. Except for *Rht2*, *Rht12*, and *Rht25*, the other genes contain UTR regions. The structures of *Rht2* and *Rht8* are relatively simple, containing only one exon. For *Rht* genes in Group I and *Rht8*, the individual exon is relatively larger, exceeding 1,871 bp, which contributes to keep integrity of protein structure, and ensures functional stability in metabolic regulation, stress response, and other processes [54]. Conservation motif analysis shows that (Fig. 2B), Group I possesses highly conserved motif structures in GRAS protein family, with 10 identical motifs. In Group Ⅱ, except for Rht4, lacking motifs 3 and 6, both Rht12 and Rht24 contain motifs 1 to 7. In Group Ⅲ, Rht25 and Rht26 share the same motifs 1 and 2, while Rht5 and Rht8 only share the same motif 1 in Group Ⅳ.

**Fig. 3:**
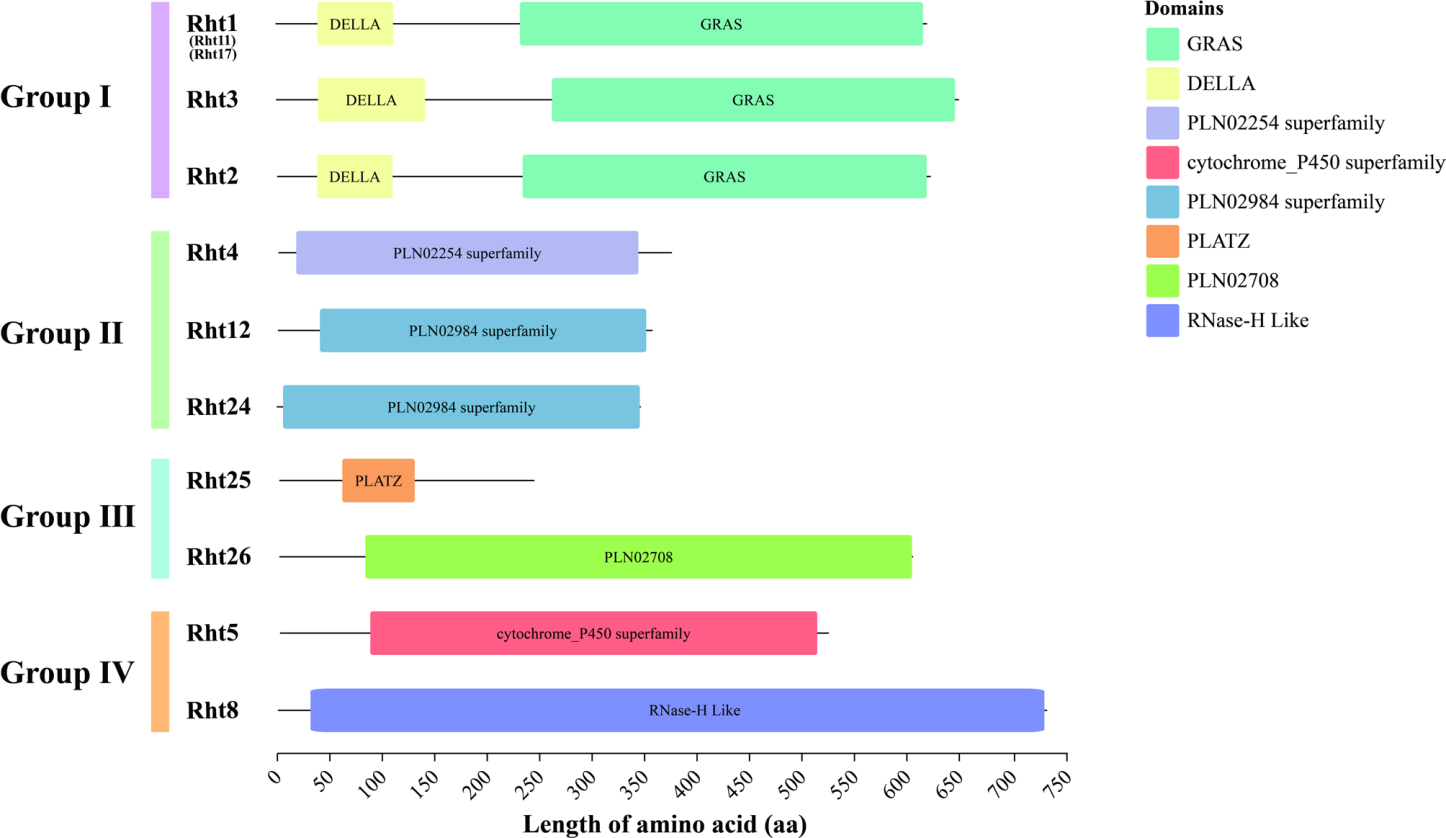
Conserved functional domains of Rht proteins

Functional domains revealed that Rht members comprised eight types (Fig. 3): GRAS, DELLA, PLN02254-Superfamily, cytochrome P450 superfamily, PLN02984-Superfamily, PLATZ, PLN02708, and RNase-H Like. Group I of Rht1, Rht2 and Rht3 possesses DELLA and GRAS domains. DELLA regulates the GA signaling pathway through SCF (Skp1-Cullin-F-box complex) mediated ubiquitination and degradation, acting as a negative regulator in signal transduction. In the Group Ⅱ, Rht4 contains PLN02254-Superfamily domain, while both Rht12 and Rht24 contain PLN02984-Superfamily domain. These two domains belong to the 2OG-Fe(II) oxygenase superfamily (2-oxoglutarate and Fe(II)-dependent oxygenase), which includes GA2ox, GA3ox, and GA20ox, catalyzing GA biosynthesis. In the Group Ⅲ, Rht25 contains PLATZ domain, and PLATZ proteins regulate seed germination, leaf cell proliferation and senescence [55, 56]. Rht26 possesses PLN02708 domain, which belongs to the pectin methylesterase family, and hydrolyze the ester bond between galacturonic acid and methyl, thereby reducing methylation levels, altering cell wall rigidity, and inducing plant dwarfism. In the Group Ⅳ, Rht5 contains P450-superfamily domain, regulating plant growth and development by synthesizing plant hormones [57], while Rht8 possesses the RNase-H like domain (Ribonuclease H-like), primarily involving in DNA replication, homologous recombination, transposition, and RNA interference [58].

**Fig. 4:**
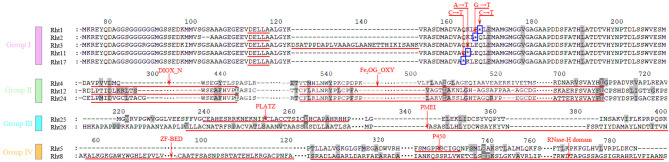
Characterization of conserved structural domains and mutation sites of Rht members Note: Top numbers indicate amino acid sites of Rht members. Red underlines indicate typical amino acid structure of Rht members, and blue boxes indicate amino acid mutation sites of Rht1, Rht2, Rht11, and Rht17

Rht amino acids revealed that all members in the Group I contain the typical amino acid sequence of DELLA proteins (Fig. 4). For Rht1 CDS, a base substitution occurs at the 190 bp position (C→T), introducing a TAG stop codon, which causes premature termination of protein. The truncated DELLA protein, lacking the C-terminal nuclear localization signal (NLS), can not be degraded by 26 S proteasome, thereby inhibiting GA signal transduction and causing plant dwarfism. Each of *Rht2*, *Rht11*, and *Rht17* has mutations at 187 bp positions (G→T), 181 bp (A→T), and 178 bp (C→T) in their CDS regions, respectively, forming TAG/TGA stop codons. The truncation mechanism is similar to that of Rht1. However, Rht3 contains a 90 bp nucleotide insertion within its CDS, which blocks GA signaling and causes plant dwarfism. In the Group Ⅱ, Rht4, Rht12, and Rht24 share conserved domains: DIOX_N (Non-haem dioxygenase in morphine synthesis N-terminal) and 2OG-FeII oxygenase. These domains bind metal cofactors (such as Fe²⁺) and catalyze oxidative reactions to modify GA precursors or activity, thereby regulating plant growth and development. In the Group III, Rht25 belongs to PLATZ family, mediating DNA binding and GA signal interaction through zinc finger domains, to regulate plant height and non-biotic stress responses. Rht26 carries PMEI domain, which inhibits pectin methylesterase activity, maintaining high pectin methylation in cell walls and regulating cell wall extensibility. In the Group Ⅳ, Rht5 belongs to the CYP450 family, which is widely involved in the synthesis of secondary metabolites (such as terpenoids and alkaloids), and the hormone modification (such as gibberellins and brassinosteroids). Rht8 carries an RNase H domain and a ZF-BED-type motif, primarily playing a central role in RNA or DNA metabolic balance and genomic stability.

### Cis-acting elements in the ***Rht*** gene promoter

**Fig. 5:**
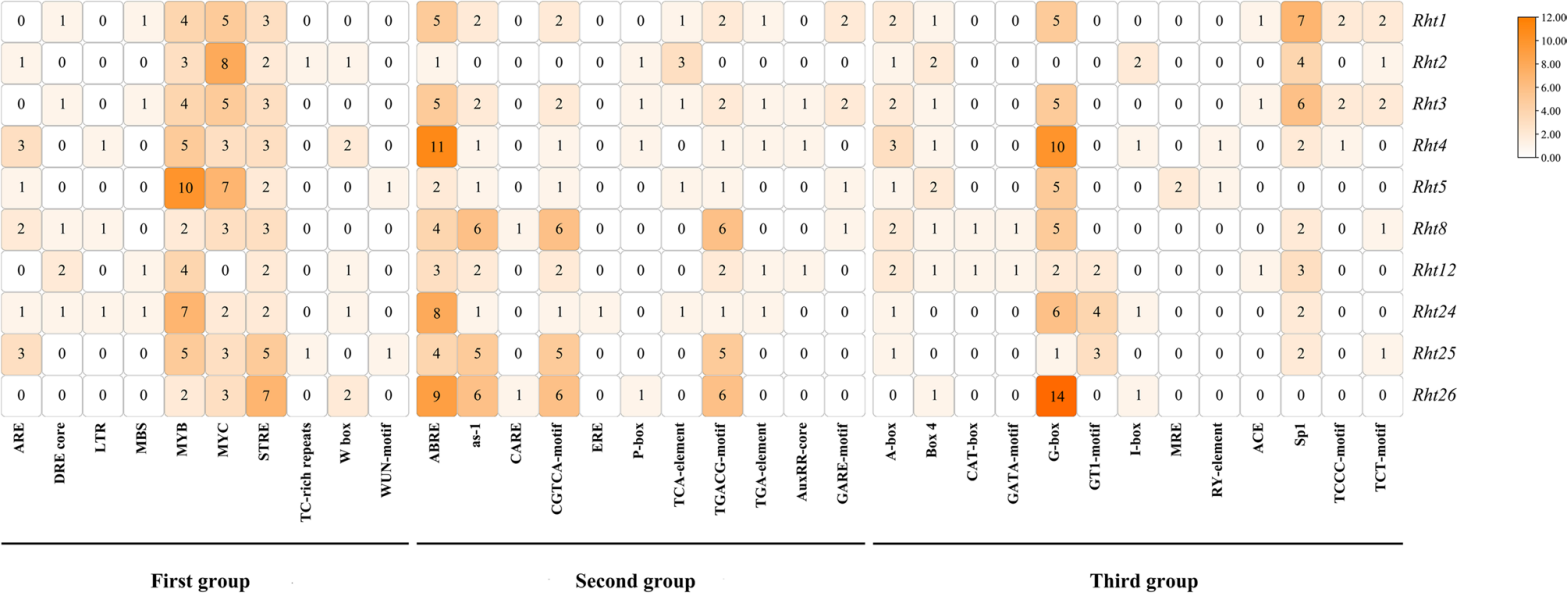
Cis-elements of promoter of *Rht* members Note: The color and number in the grid Figure indicate the counts of different cis-elements in *Rht* promoters. The first group represents abiotic and biotic stresses, the second group represents phytohormone responsive, and the third group represents plant growth and development. The predicted types and functions of cis-elements of *Rht* promoters were listed in Table S4

*Rht* gene promoter contains 34 cis-acting elements, which are divided into three groups (Fig. 5). The first group includes 10 types, primarily responding to biotic and abiotic stresses, such as antioxidant (ARE), dehydration (DRE core), low temperature (LTR), drought (MBS), heat shock (STRE), defense stress (TC-rich repeats), and fungal induction (W box). The second group includes 11 types associated with plant hormone induction, such as abscisic acid (ABRE), salicylic acid (as-1), gibberellin (CARE), methyl jasmonate (CGTCA-motif and TGACG-motif), ethylene (ERE), salicylic acid (TCA-element), and auxin (TGA-element). The third group includes 13 types associated with plant growth and development, such as light response (A-box, Box 4, G-box, GT1-motif, I-box, Sp1, ace, GATA-motif, TCCC-motif, and TCT-motif), meristem-specific expression (CAT-box), and seed development (RY-element). These results suggest that *Rht* genes may play important roles in plant light induction, abscisic acid, salicylic acid, and methyl jasmonate regulation, as well as drought stress resistance.

### Collinearity analysis of *Rht* members

**Fig. 6:**
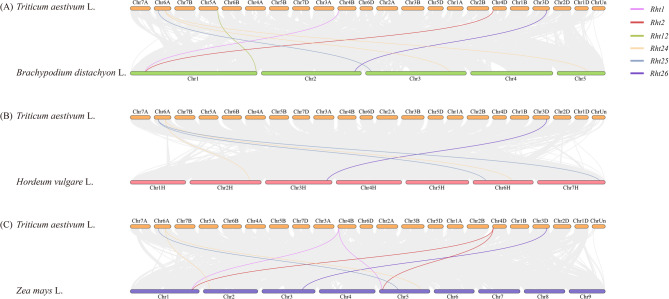
Collinearity analysis of *Rht* members between wheat and *Brachypodium distachyum*, barley, and maize Note: **A** is between wheat and Brachypodium distachyum. **B** is between wheat and barley. **C** is between wheat and maize. Lines in peach pink, orange-red, green, pink, dark blue, and purple represent the *Rht1*, *Rht2*, *Rht12*, *Rht24*, *Rht25*, and *Rht26* genes, respectively. Collinearity associations were analyzed by TBtools v2.056 software. Detailed information of *Rht* genes in wheat, *Brachypodium distachyum*, barley, and maize was listed in Table S5

Gramineae plants share a common ancestor, and collinear gene pairs between different species exhibit similar functions [59]. There are seven pairs of collinear gene pairs between wheat and Brachypodium, each of *Rht1*, *Rht2*, *Rht12*, *Rht25*, and *Rht26* has one collinear gene, with 59, 52, 40, 77, 108 pairs of anchor genes, respectively. *Rht24* has two pairs of collinear genes, with 72 and 15 pairs of anchor genes (Fig. 6, Appendix Table S5). There are five pairs of collinear genes between wheat and barley, *Rht26* has one pair with 651 pairs of anchor genes, and each of *Rht24* and *Rht25* has two pairs. There are eight pairs of collinear genes between wheat and maize, each of *Rht1*, *Rht2*, and *Rht24* has two pairs, and *Rht1* has 165 pairs of anchor genes. Each of *Rht25* and *Rht26* has one pair. Among these genes, *Rht24*, *Rht25*, and *Rht26* exhibit collinearity in Brachypodium, barley, and maize, indicating that the functions of three genes are highly conserved across different species. The Ka/Ks ratios of these gene pairs were all less than 1, with the exception of the collinearity index between wheat and barley, specifically for the pairing of *Rht25* and its putative ortholog MOREX.r3.7HG0738060. This finding indicates *Rht* genes are subject to purifying selection and their functions have been highly conserved during evolution.

**Fig. 7:**
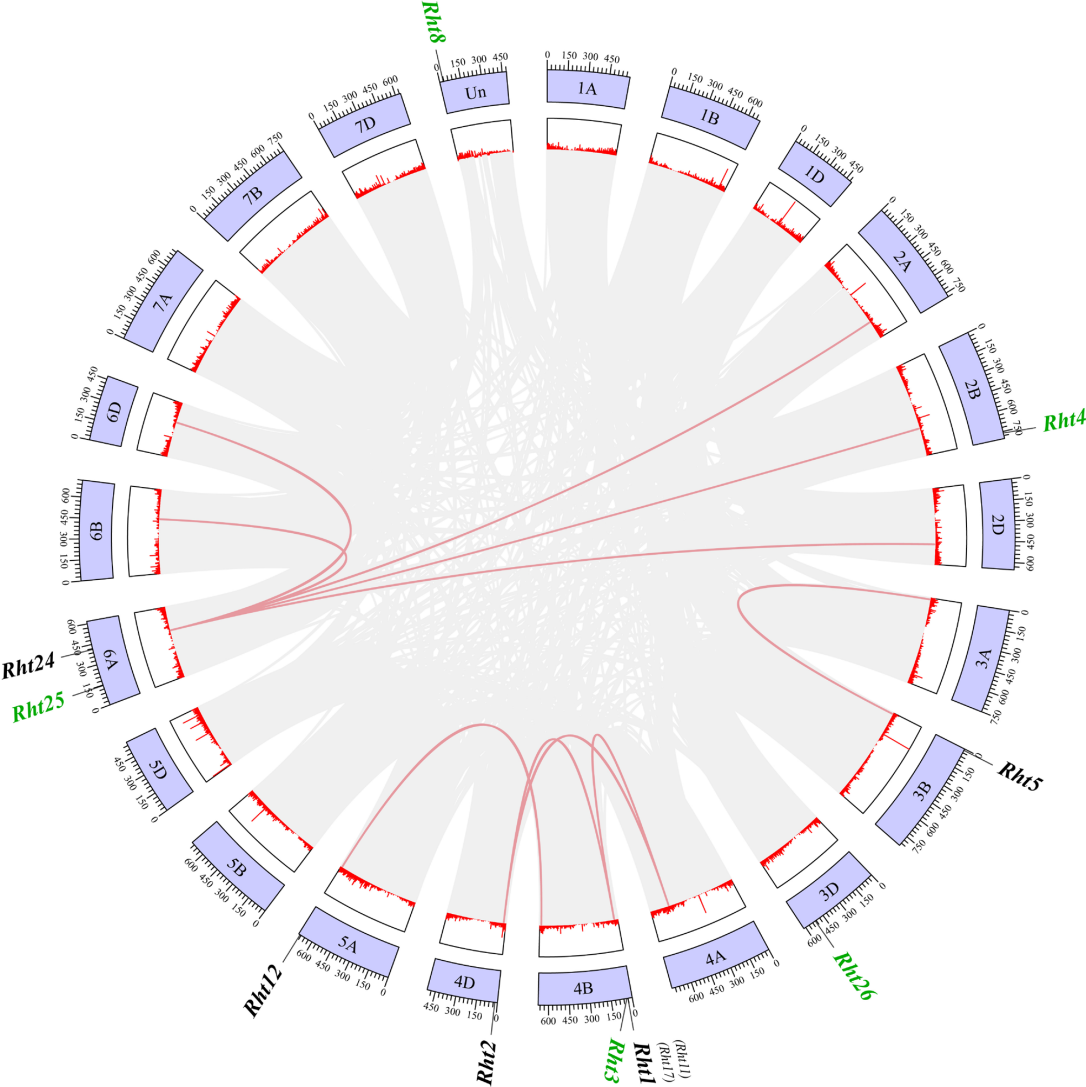
Collinearity analysis of *Rht* members in wheat genomes Note: Green genes represent no collinearity relationships in the wheat genome. Detailed information of *Rht* members was listed in Table S2

In the wheat genome, collinearity relationships were found for the *Rht1*, *Rht2*, *Rht5*, *Rht12*, and *Rht24* genes (Fig. 7), each of *Rht1* and *Rht2* has two pairs of collinear genes, each of *Rht12* and *Rht5* has one pair, and *Rht24* has five pairs of collinear genes, with 1253 pairs of anchor genes. *Rht1* and *Rht2* are located on different chromosomes and exhibit a colinear relationship, suggesting that they likely originated from an chromosomal segment duplication event [48]. The low Ka/Ks ratio of 0.078 value suggests that both genes undergo intense purifying selection after duplication, thereby maintaining high functional conservation.

### *Rht* protein interaction analysis

**Fig. 8:**
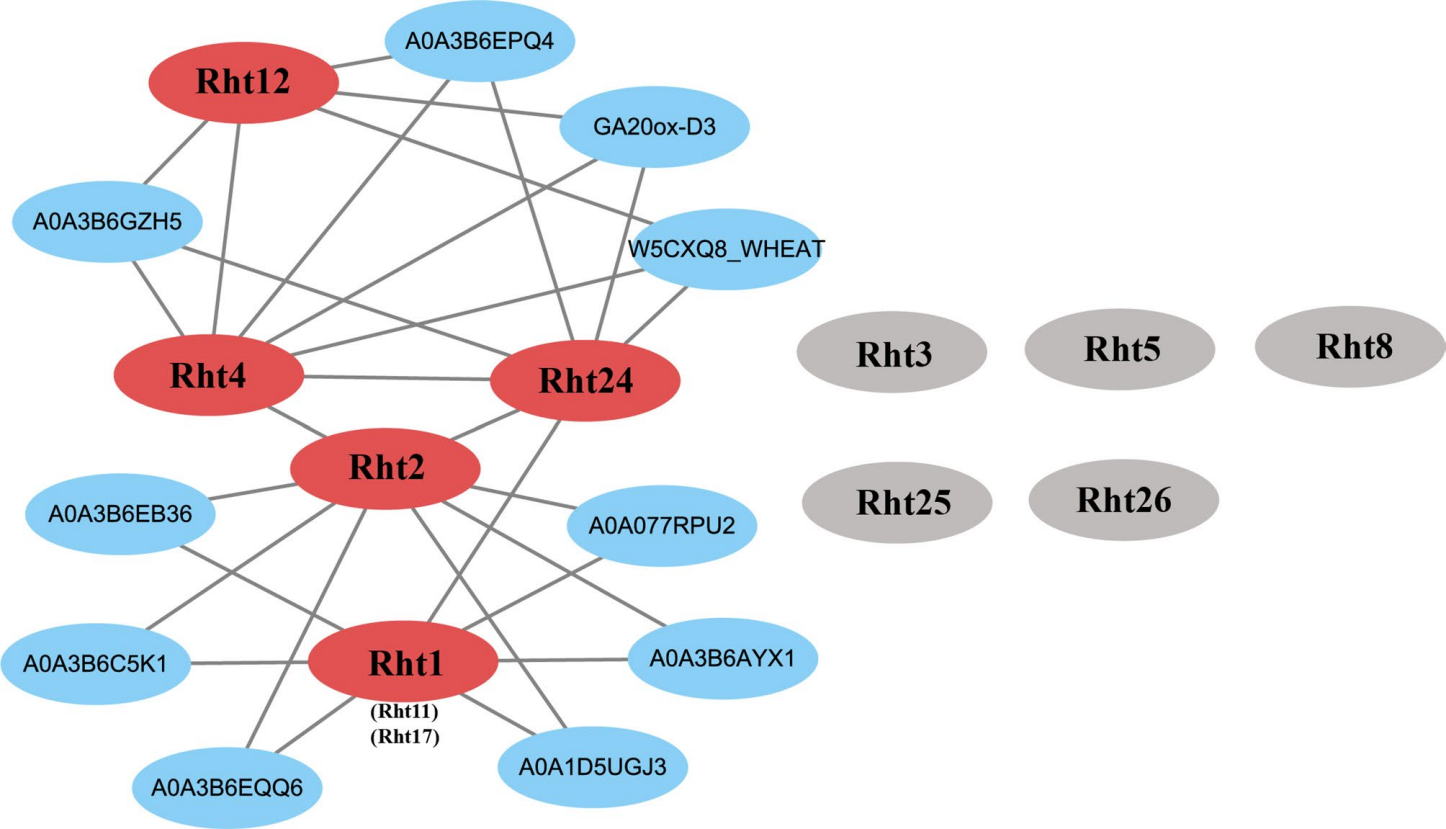
Interaction network of 10 Rht proteins Note: The interaction network of Rht proteins was predicted using the STRING database, and the interaction network model was optimized and visualized by using Cytoscape 3.9.1 software

Rht protein interaction network indicates that Rht1, Rht2, Rht4, Rht12, and Rht24 occupy pivotal positions. Rht4 interacts with multiple Rht proteins, including Rht2, Rht12, and Rht24, while Rht1 shows a specific interaction with Rht24 (Fig. 8). GA20ox-D3, a rate-limiting enzyme in the gibberellin biosynthesis pathway, interacts with Rht4, Rht12, and Rht24. This suggests GA20ox-D3 may regulate gibberellin synthesis, thereby participating in the regulation of key development processes, such as plant height, seed germination, and flowering time [60, 61]. Rht4, Rht12, and Rht24 interact with multiple proteins, which belong to the iron/ascorbate-dependent oxidoreductase family, such as A0A3B6EPQ4, A0A3B6GZH5, W5CXQ8_WHEAT. These proteins play central roles in reactive oxygen species homeostasis, antioxidant responses, and metabolic regulation, being extensively involved in plant immune responses. Basic Helix-Loop-Helix (BHLH) transcription factor-related proteins, such as A0A3B6AYX1, A0A1D5UGJ3, and A0A3B6C5K1), F-box family proteins, such as A0A077RPU2, A0A3B6EB36, and A0A3B6EQ06, were also identified as key interaction partners of Rht1 and Rht2. BHLH proteins respond to abiotic stresses, such as drought and low temperature, while F-box proteins play a crucial role in regulating the stability of stress-related proteins. These interaction networks reveal that the proteins collectively participate in regulating plant development and stress response processes.

### GO annotation analysis of *Rht* members

**Fig. 9:**
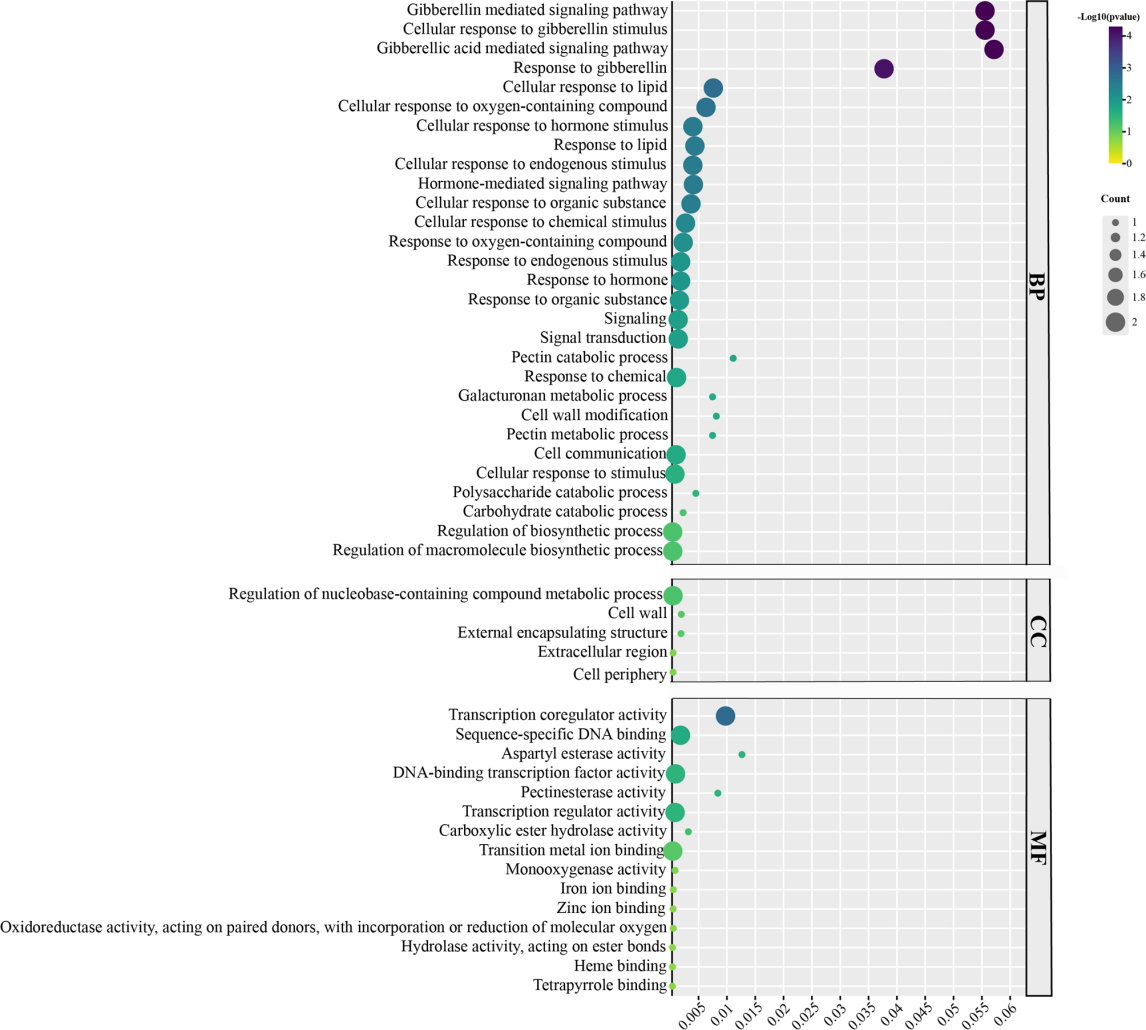
GO annotation analysis of *Rht* members Note: The dot size in the Figure represents the number of genes with corresponding GO annotations, and the value of the abscissa axis represents the enrichment degree, the larger value, the higher enrichment degree. BP is biological process, CC is cellular component, MF is molecular function

GO enrichment analysis of 10 *Rht* genes revealed significant enrichment in gibberellin-related signaling pathways within the Biological Process (BP) category (Fig. 9). “Gibberellin-mediated signaling pathway” and “Cellular response to gibberellin stimulus” were most prominent, with enrichment analysis Q-values reaching 5.08 × 10^⁻⁵^, indicating that *Rht* genes play a central role in gibberellin signaling and response processes. Regarding cellular components (CC), *Rht* genes are localized to the cell wall, external encapsulating structures, and the cell periphery, suggesting *Rht* genes may participate in the synthesis and modification of cell wall polysaccharides, thereby regulating cell wall structure and growth development. For molecular function (MF), *Rht* genes showed significant enrichment in transcription coregulator activity, specific DNA binding, and carboxylic ester hydrolase activity, suggesting these genes may regulate specific gene expression as transcription coactivators or DNA-binding proteins. (Fig. 9).

### miRNA analysis targeting *Rht* genes

**Fig. 10:**
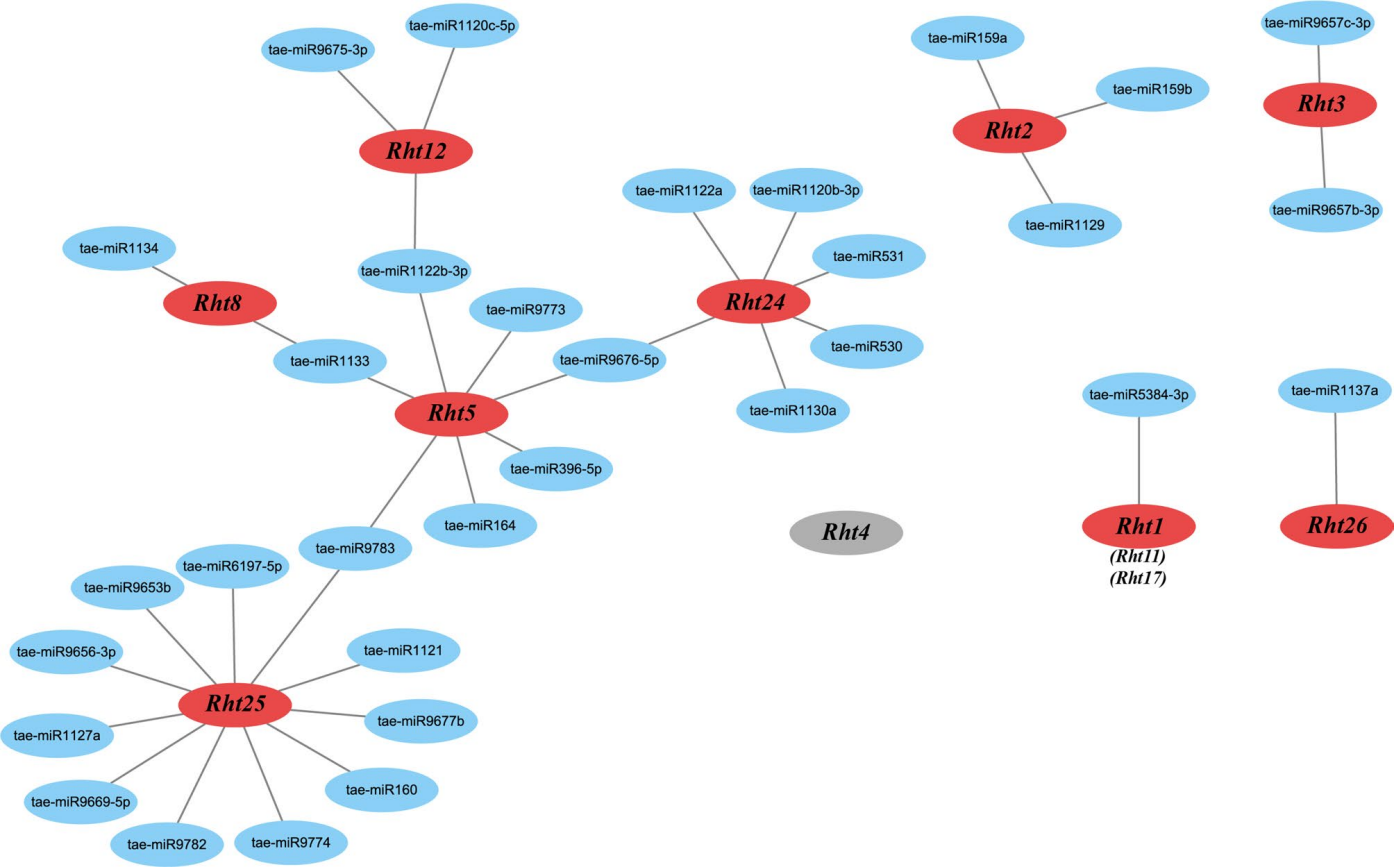
Interaction network between *Rht* members and target miRNA molecules Note: The red ovals represent 9 *Rht* genes, gray indicates *Rht4* member without targeting to miRNA molecules, and blue ovals represent miRNA molecules that target to each gene of *Rht* members

A total of 32 miRNA molecules were identified, ranging in length from 19 to 24 bp, targeting nine *Rht* genes in wheat (Fig. 10). *Rht5* interacts with multiple miRNAs, including tae-miR1133, tae-miR1122b-3p, tae-miR9773, tae-miR9676-5p, tae-miR164, and tae-miR396-5p, and shares miRNAs with other *Rht* genes, such as *Rht8*,* Rht12*,* Rht24*,* and Rht25.* These findings support *Rht5*’s role as a central hub. miRNAs targeting *Rht5* are located within the region of CDS and 3’ UTR, showing high-confidence binding sites (E-value ≤ 5), which suggest that *Rht5* occupies a central node to influence downstream signaling and metabolic network, and probably regulate wheat growth and development. To validate the target miRNA function of *Rht5*, further experiments could involve constructing transgenic wheat with miRNA overexpressing or target mimics. These approaches can examine the effects of increased or blocked expression of specific miRNAs on the transcriptional level of *Rht5*, and integrate observations of key agronomic trait changes of wheat induced by miRNA manipulation.

### Expression patterns of *Rht* genes

#### Expression specificity in different tissues of wheat

**Fig. 11:**
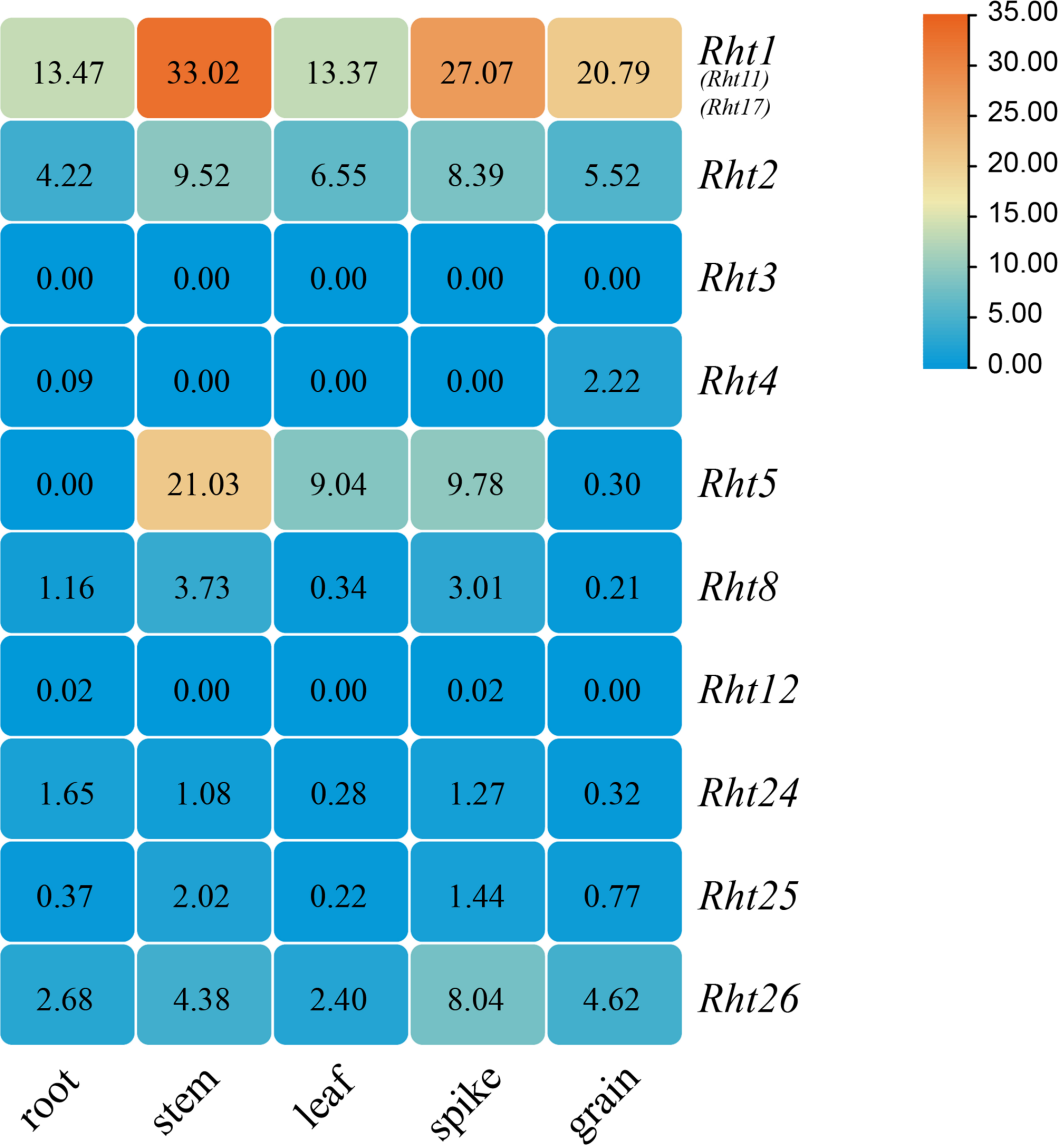
Expression analysis of *Rht* genes in different tissues of wheat Note: The expression data of target genes was obtained from Wheatomics database (wheat variety Chinese Spring). *Rht3* gene was obtained from dwarfing variety Tom Thumb (the germplasm of Tibet, China), and its expression data was not included in this database. The numbers in the grid Figure represent the expression level of *Rht* genes with FPKM values. Red part of the bar indicates the highest expression level, while the lowest expression level with the blue part

The expression of *Rht* genes varies significantly across different tissues. The expression characteristics of *Rht3* gene, being derived from the dwarf variety Tom Thumb, has not been documented in the Chinese Spring genome, with a zero expression level (Fig. 11). In contrast, *Rht4*, *Rht8*, *Rht12*, *Rht24*, and *Rht25* showed no significant expression in root, stem, leaf, spike, and grain, while *Rht1*, *Rht2*, and *Rht26* exhibited relatively high expression levels in the five tissues. Compared to other genes, *Rht1* exhibited significantly higher expression levels than other members in the tissues, while *Rht5* showed higher expression levels only in stems, leaves, and spikes.

#### Expression patterns under abiotic stress and hormone treatments

**Fig. 12:**
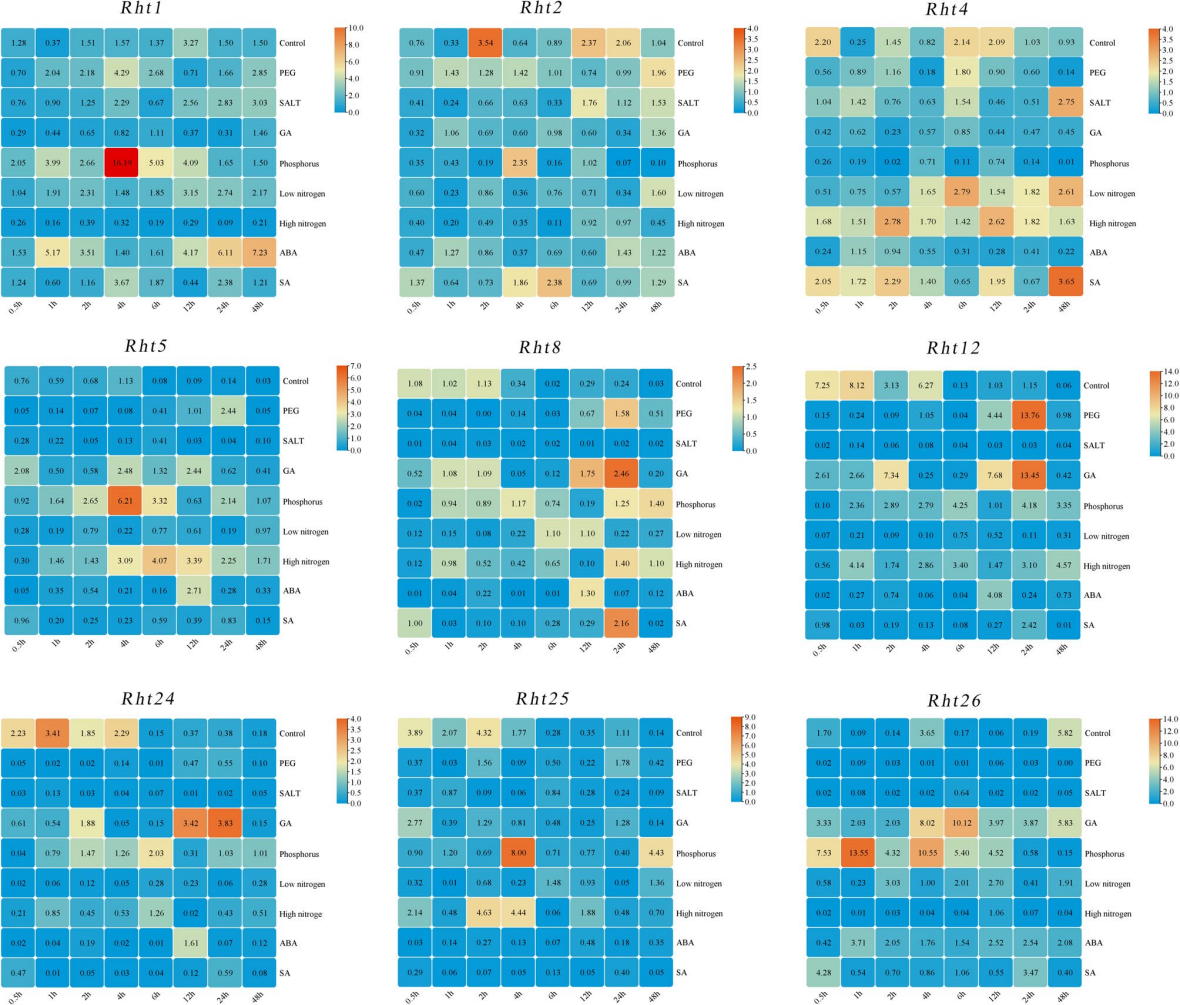
Expression patterns of *Rht* genes under different treatments Note: Stress treatments include 20% PEG_6000_ (W/V), 150 mM/L NaCl solution, 200 µmol/L GA_3_ solution, 10 mg/L phosphorus solution, ammonium nitrate solution (0.5 mM/L and 2.5 mM/L for low nitrogen and high nitrogen, respectively), 1000 µmol/L ABA solution, and 0.6 mmol/L SA solution. The numbers in the grid Figure represent the relative expression level of *Rht* genes. Red part of the bar indicates the highest expression level, while the lowest expression level with the blue part. Over 95% of the data achieved a highly significant level (FDR < 0.05) and all corrected p-values are provided in Appendix S8

Compared with the control, under PEG treatment, *Rht1* was continuously upregulated from 1 h to 6 h, with the highest expression level at 4 h, *Rht5* and *Rht8* showed the highest expression level at 24 h., and *Rht12* was significantly upregulated at 12 h and 24 h, maximum log_2_FC value increased to 3.66 (Fig. 12). Under salt treatment, *Rht1* and *Rht2* showed upregulation from 12 h to 48 h, with the highest expression level at 48 h for *Rht4*, while *Rht8*, *Rht12*, *Rht24* and *Rht26* showed downregulation. Under GA induction, *Rht1* exhibited downregulation, *Rht5* showed significant upregulation from 4 h to 12 h, *Rht8*, *Rht12*, and *Rht24* maintained upregulation from 12 h to 24 h, all reaching peak expression at 24 h, while *Rht26* exhibited continuous upregulation from 0.5 h to 48 h. Under phosphate stress, *Rht1*, *Rht2*, *Rht5* and *Rht25* showed significant upregulation at 4 h, with *Rht26* reaching peak expression at 1 h. *Rht8* showed higher upregulation from 24 h to 48 h, while *Rht4* showed downregulation. For nitrogen stress, under low nitrogen conditions, *Rht4* showed higher expression levels at 6 h and 48 h, with upregulation of *Rht8* from 6 h to 12 h, and *Rht2* showed continuous downregulation from 0.5 h to 24 h. Under high nitrogen conditions, *Rht4* showed significant upregulation, *Rht5* showed continuous upregulation from 1 h to 48 h, as well as *Rht8* from 24 h to 48 h. *Rht25* had higher expression levels from 2 h to 4 h, and both *Rht1* and *Rht2* showed downregulation. For abscisic acid treatment, *Rht1* exhibited relatively high expression levels between 12 h and 48 h, while *Rht5* reached its peak at 12 h, which was 30 times higher than control. *Rht8*, *Rht12*, and *Rht24* were significantly upregulated only at 12 h, while *Rht25* showed significant downregulation from 0.5 h to 6 h. For salicylic acid treatment, *Rht1* was significantly upregulated at 4 h and 24 h, as well as *Rht2* from 4 h to 6 h. *Rht4* showed peaking upregulation at 48 h, *Rht8* showed continuous upregulation from 6 h to 24 h, and *Rht25* showed downregulation. In summary, under PEG treatment, *Rht12* showed the most significant response, and under GA induction, *Rht8*, *Rht12*,* Rht24*, and *Rht26* showed high-level upregulation. Additionally, *Rht1*, *Rht5*, *Rht25*, and *Rht26* showed high-level upregulation under phosphate treatment, with *Rht1* exhibiting the greatest response. Under salicylic acid treatment, the expression level of *Rht4* significantly increased. These results indicate that the expression of different *Rht* genes exhibits highly specific response patterns to specific treatments, and these dynamic changes may be closely related to the regulation of specific agronomic traits. (Appendix Table S11)

#### Dwarf gene identification and genetic analysis of different wheat varieties

In two crop seasons (Table 3), PH and MSSGN showed significant difference between both years, while the varieties exhibited greatly significant difference for traits of PH, MSSL, MSSGN, TKW and GY. However, there were no significant interaction effect between year and variety. Based on the information of plant height and yield-related traits of 47 varieties in two years, the cluster analysis showed that 47 varieties were divided into seven groups with yield-related traits, Group Ⅵ and Group Ⅶ exhibited high yields, exceeding 10,050 kg·ha⁻¹, mainly due to superior yield traits, such as MSSGN and TKW, Group I and Group II exhibited low yields, below 6600 kg·ha⁻¹, due to poor TKW. For plant height, 47 varieties were divided into six groups, Group I and Group II exhibited dwarf status, with plant height below 62 cm, while Group V and Group Ⅵ were taller, reaching over 72 cm (Fig. S1).

**Table 3:** Variance analysis (ANOVA) on agronomy traits of wheat

MS	df	PH (cm)	MSSL (cm)	MSSGN (No.)	TKW (g)	GY (kg·ha^−1^)
Year (Y)	1	63.67*	44.24	11.12*	0.13	3529.45
Variety (V)	46	216.71**	21.81	136.55**	53.99**	1420.74**
Y*V	46	0.78	19.46	1.35	9.78	780.70
Replication	10	12.66	12.62	11.520**	19.31	2387.05
Error	186	9.01	19.51	1.37	13.80	5140.51
F	96	11.789**	1.062	48.85**	2.27**	1338.36**

Note: df is degrees of freedom, PH is plant height, MSSL is main stem spike length, MSSGN is main stem spike grain number, TKW is thousand kernel weight, GY is grain yield. * and** indicate significant difference at the *P* < 0.05 and *P* < 0.01 levels, respectively

In the allele frequencies of 47 wheat varieties, *Rht24* exhibited the highest distribution frequency of 91.5%, as well as *Rht12*, *Rht8*(*Rht2*), *Rht4*, *Rht1*, *Rht5*, showing 72.3%, 53.2%, 38.3%, 17.0%, 8.5%, repectively. Linkage disequilibrium analysis among loci revealed that the r² value was less than 0.1, indicating no strong linkage disequilibrium among *Rht* genes loci. (Appendix Table S10)

**Fig. 13:**
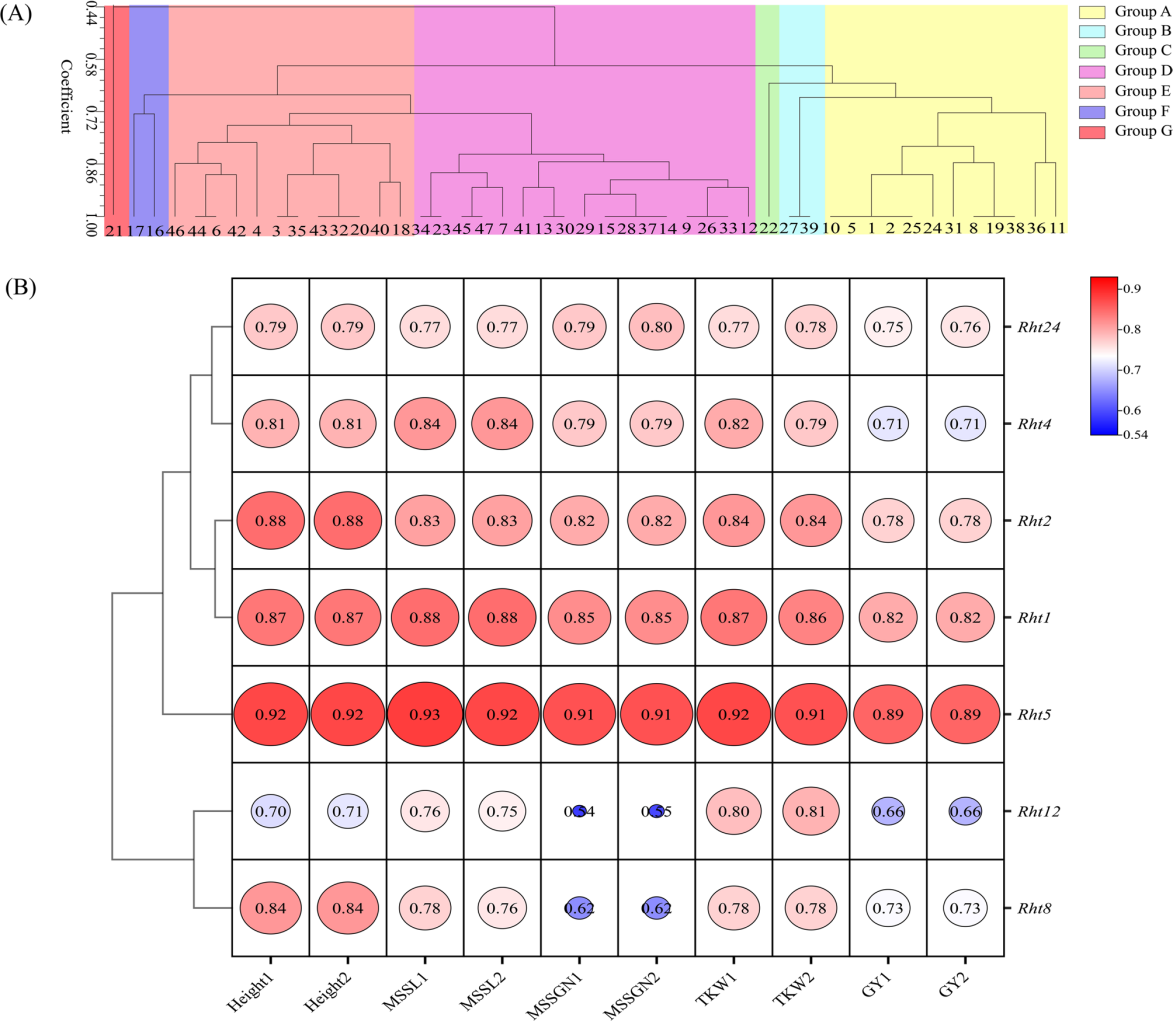
Gray correlation analysis between distribution types of *Rht* genes and agronomic traits in different wheat varieties Note: **A** Cluster analysis of 47 wheat varieties with different distribution types of *Rht* genes. **B** Gray correlation analysis between distribution types of *Rht* genes and agronomic traits. MSSL is main stem spike length, MSSGN is main stem spike grain number, TKW is thousand kernel weight, GY is grain yield. 1 and 2 represent data collected in 2023 and 2024, respectively. Circle size and value represent the correlation degree, the larger circle and value, the stronger correlation degree

47 wheat varieties were classified into seven groups (Fig. 13A and Appendix Table S10). In Group A, plant height of variety VICT0 was only 55 cm, while the other varieties ranged from 69 to 78 cm. VICT0 had the lowest grain yield with *Rht1* and *Rht2*, while mostly other varieties carried *Rht12* or *Rht24* in addition to *Rht1* and *Rht2*. Group D had shorter plant height, with a range of 60–69 cm, while grain yield reached 7950–9900 kg·ha⁻¹, most varieties in Group D carried *Rht4* and *Rht8*, indicating that the combination of *Rht4* and *Rht8* may simultaneously optimize plant architecture and yield potential. In Group E, except for LH7, LY502, ZM7698, the remaining varieties had plant heights of 63–73 cm, and grain yield of 9150–11,250 kg·ha⁻¹, which were higher than that in Group D. Compared with Group E, most varieties in Group D additionally contained *Rht4*, and their plant height and grain yield were generally lower, suggesting that *Rht4* may overly inhibit stem development, thereby partially offsetting a yield-enhancing effect. In Group E, the combination of *Rht8*, *Rht12*, and *Rht24* maintained a semi-dwarf plant type while synergistically regulating spike development, to achieve higher grain yield.

A grey correlation analysis showed (Fig. 13B) that *Rht5* exhibited extremely high correlations with plant height and yield-related traits, with all correlation coefficients (r) above 0.89, indicating that *Rht5* may directly regulate development of plant height and yield traits. Both *Rht1* and *Rht2* showed strong correlations with plant height, MSSL, MSSGN, and TKW, indicating that the two genes may synergistically regulate spike development and key yield traits. *Rht8* and *Rht12* exhibited strong correlations with plant height and TKW, respectively, confirming that *Rht8* plays an important role on plant height regulation, and *Rht12* may be involved in grain filling and development. *Rht4* showed strong correlations with plant height, MSSL and TKW, implying *Rht4* influence on spike development while controlling plant height. To confirm their genetic effects on target traits, the following experiments can use standard association analysis models as multiple linear regression in larger independent breeding populations, to account for population structure and kinship relationships of germplasms with *Rht* genes.

## Discussion

### Evolution and characteristics of *Rht* genes based on comparative genomics

The amino acid lengths of 10 *Rht* genes range from 243 to 808 amino acids, DELLA proteins encoded by Rht1 and Rht2 act as core inhibitory factors in the GA signaling pathway. In contrast, truncated proteins Rht11 and Rht17 increase sensitivity to stress factors, such as drought and salinity [62]. The instability indexes of Rht4, Rht5, and Rht12 are below 40, indicating relatively stable proteins, ensuring GA levels to maintain a sustained influence on plant height. Rht5 and Rht26 were localized in the vacuole and chloroplast, respectively, which was speculated that they maintained the endogenous hormone homeostasis through the vacuole region, enhanced interactions with mitochondrial or chloroplast membranes, and participated in energy metabolism regulation [63, 64].

The 10 Rht proteins were divided into four families (I–Ⅳ). In Group I, Rht1 (Rht11, Rht17), Rht2, and Rht3 were found to contain DELLA and GRAS domains. GRAS domain has five highly conserved motifs, namely LHRI, VHIID, LHRII, PFYRE, and SAW [65]. Among these, DELLA protein has a GA-sensing domain in N-terminal region, which binds to the GA receptor GID1, and interacts with various proteins to inhibit their activity, making plants insensitive to GA to reduce plant height [66]. In Group Ⅱ, Rht4, Rht12, and Rht24 possessed two conserved domains, DIOX_N and 2OG-FeII oxygenase. Mutations in these domains block GA biosynthesis, reduce GA content, and lower plant height. In Group Ⅲ, Rht25 encoded a transcription factor belonging to PLATZ family (Plant AT-rich sequence and Zinc-binding), containing two conserved domains: C.H.C.C.C.C.HH.H and C.C…. C.C, being reported to involve in interaction between growth regulating factor and DELLA during plant growth [38]. Rht26 encoded the PMEI51, a homologue of pectin methylesterase inhibitor 51. *Rht26* gene was localized in the cell wall, external envelope, and periplasm, participating in cell wall modification and pectin metabolism in GO enrichment analysis. In rice, overexpression of *OsPMEI28* led to abnormal cell elongation in straw tissues, resulting in dwarf transgenic lines [55]. Group Ⅳ included the dwarf genes Rht5 and Rht8. Rht5 belongs to P450 family, which directly regulates endogenous brassinosteroid (BR) synthesis levels by catalyzing the C6 oxidation reaction, thereby influencing cell elongation, stress resistance, and plant architecture development [67]. Through inhibiting BR synthesis or signal transduction, Rht5 can reduce the expression of target genes related to cell wall relaxation, and synergistically inhibit stem elongation with GA. Rht8’s unique advantage is that it does not disrupt the integrity of GA signaling pathway, retaining the GA sensitivity of coleoptile cells, and ensuring seedling emergence capacity and grain filling potential [48].

*Rht* promoters can be classified into three categories: growth and development, hormone synthesis, and biotic and abiotic stress. The promoter regions of 10 *Rht* genes were identified 52 ABRE elements, which is higher than that of other wheat gene families reported, such as *TaCOI*, *TaSPL*, and *TaOPR*, implying that ABA signaling pathway involves in a crucial mechanism of *Rht* family [68–70]. In addition, the promoters of *Rht* members are rich with stress response element binding sites, such as MYB, MYC, and STRE, which involved in hormone-mediated signaling processes, such as GA, JA, and ABA [70]. Notably, the stress response element STRE is often colocalized with ABRE [71], suggesting that *Rht* may respond to environmental stress signals via STRE. To clarify the biological function of element enrichment, the promoter-GUS reporter system can be employed to quantitatively analyze transcriptional activity, as well as in vivo binding between transcription factors and these cis-elements, through ChIP-qPCR technique.

#### Expression dynamics of *Rht* genes

Expression patterns of *Rht* genes vary significantly in different tissues and different stress conditions. After GA treatment, *Rht26* started to respond at 0.5 h and reached its peak at 6 h, this is speculated that *Rht26* catalyzes the oxidation reaction of GA precursors, and elevates endogenous GA levels, which is consistent with previous study [72, 73]. *Rht12* and *Rht24* exhibited high expression levels between 12 h and 24 h, which may hydroxylate GA4 of active gibberellin into inactive GA34, thereby inactivating GA signal and activating ROS scavenging system mediated by *SOD1* and *SOD2* genes [74, 75].

Only under high nitrogen conditions, *Rht5* was upregulated from 4 h to 48 h. As *Rht5* belongs to P450 family, it was speculated to catalyze the synthesis of secondary metabolites, such as phenols and flavonoids, enhance nitrogen uptake or transport of roots [76]. DELLA protein encoded by *Rht1* not only regulates GA signaling pathway but also serves as a key factor responding multiple stress signals [77]. Under ABA treatment, *Rht1* gene exhibited sustained high expression levels from 12 h to 48 h, confirming that *Rht1* actively participates in the ABA-mediated stress mechanism. *Rht1* also involves in stabilizing the antioxidant enzymes activity in plant, enhancing the drought tolerance through the oxidative stress defense system [78]. *Rht26* inhibits pectin methylesterase activity, reduces the methylation level of pectin in the cell wall, and affects cell wall structure [79], following SA treatment, *Rht26* expression exhibited fluctuating changes, suggesting that *Rht26* may synergize with SA signaling mechanism to activate glucosyl transferase, such as gibberellin stimulated-like, promote the deposition of β−1,3-glucan at plasmodesmata, and thereby hindering pathogen invasion. In the future, the role of *Rht* on hormone induced pathways and environmental stress can be validated by enzyme activity assays, CRISPR knockouts (overexpression) lines, PMEI activity tests and cell mechanical analysis.

#### Synergistic regulatory effects of allele stacking on agronomic traits of wheat

27 dwarfing genes exhibit a distinct polyploid-specific distribution during species evolution. Diploid wheat contains only *Rht27*, tetraploid wheat contains seven genes, such as *Rht9*, *Rht14*, *Rht15*, *Rht16*, *Rht18*, *Rht19*, and *Rht22*, and hexaploid wheat contains 19 genes, such as *Rht1*, *Rht8*, *Rht10*, *Rht13*, *Rht17*, *Rht20*, *Rht21*, *Rht23*, and *Rht26*. The dwarfing effects of different *Rht* genes exhibit significant differences, for example, *Rht1* and *Rht2* reduce plant height by 20% [2], *Rht3* and *Rht11* exhibit a 64% dwarfing effect, and *Rht10* for extreme dwarfism by increasing gene copies, with a stem-reducing effect exceeding 64% [21]. In this study, the variety VICT0 only carries *Rht1* and *Rht2*, and its TKW is significantly lower than that of other varieties in the same group, while other varieties contain *Rht12* or *Rht24*, in addition to *Rht1* and *Rht2*, exhibiting a significant increase in TKW, indicating that the dwarfing genes are associated with negative effects, and *Rht12* or *Rht24* could compensate for the negative effect of *Rht1* and *Rht2* on TKW. Wheat varieties with *Rht4* in group D have a significantly lower TKW than those in group E without *Rht4*, confirming the negative effect of *Rht4*. Yield traits cluster revealed that the varieties in high-yield Group 6 and Group 7 carry *Rht24*, confirming that *Rht2*4 may specifically increase yield and maintain semi-dwarf characteristics [49], and *Rht24* is a core genetic factor in the high-yield formation. *Rht8* regulates *GA13ox* and *GA20ox-2* genes related to GA synthesis, reducing GA_3_ activity, maintaining GA_4_ levels, and enhancing germination ability [6, 37]. The genotype combination of *Rht8*, *Rht12*, and *Rht24* occurred at a frequency of 43.75%. Although the combination exhibited weaker stem-shortening ability compared to other genotypes, it demonstrated higher TKW and GY. This suggests that *Rht8* + *Rht12* + *Rht24* probably contributes to spike development and grain filling. It was reported that wheat variety with combination of *Rht-D1b* + *Rht8* + *Rht12* + *Rht24* showed higher plant height, comparing to that of *Rht-D1b* + *Rht12* + *Rht24*, and *Rht8* and *Rht12* exhibited negative-interaction mechanisms, fail to enhance dwarfing effect [80], which aligned with the results in this study. Here, some varieties with *Rht8* + *Rht12* + *Rht24* achieved yields ranging from 9150 to 11,250 kg·ha⁻¹, suggesting the potential application value of these genes combination.

Modern wheat variety showed polygenic combination of *Rht* dwarf genes, due to their hybrid parents with different dwarf genes. JM22 contains *Rht2* + *Rht12*, the hybrid variety JM23 (Yu Mai 34×JM22) inherits the *Rht2* + *Rht12* dwarf gene combination. JN17 contains *Rht2* + *Rht24*, and the hybrid variety JM44 (954072× JN17) contains the gene combination. This polygenic combination avoids the negative effects caused by excessive regulation of a single dwarf gene, providing a genetic foundation for high-grain breeding [33, 81]. Among 47 varieties, *Rht1* gene is mainly distributed in the varieties from Henan, Shaanxi, and Hebei province, mostly due to their parental materials of BNAK58, Zhoumai16, etc., which were used to hybridize for excellent breed with *Rht1* and *Rht24*. *Rht2* gene is mostly distributed in the varieties from Shandong province, mainly due to their parental materials of YN19 and JM22, etc., wheat Yannong and Jimai mostly contain *Rht2* + *Rht24*, while Shannong series mostly contain *Rht2* + *Rht8* genes. Dwarfing effect of *Rht1* + *Rht2* was superior to that of *Rht12* + *Rht24* combination, as was shown in variety VICT0. The combination of *Rht8*, *Rht12* and *Rht24* achieved high grain yield of 9150–11,250 kg·ha^−1^ in Group E of wheat varieties, while the *Rht12* + *Rht24* only with grain yield of 7230–8850 kg·ha^−1^, which was speculated that *Rht8* enhanced sensitivity to GA, improved tillering capacity and environmental adaptability for high yield, under *Rht12* + *Rht24* double-gene dwarfing effect.

It was reported that the polygenic combination mechanisms conferred desirable plant traits for new varieties. The combination of *Rht12* with *Rht8* or *Rht24* constituted a core combination of dwarfing genes in wheat breeding [22]. In the Group 3 and Group 4 of plant height, varieties XN9871, YN999, and ZM36 contained a core combination of *Rht8* + *Rht12* + *Rht24*, with plant height ranging from 63 to 73 cm. It was confirmed that *Rht4* + *Rht8* was introduced to wheat, not only reducing plant height but also significantly improving key agronomic traits, such as TKW and grain yield, effectively mitigating the negative effects of the single *Rht4* gene [82]. In group D, varieties SN28 and ZM379, which contained *Rht4* + *Rht8* combination, achieved plant height of 60–69 cm, and significantly increased grain yields of 9150–11,130 kg·ha^−1^. However, *Rht4* + *Rht1* did not have an additive effect on plant height, the combination can increase spikelet number, grain number, grain yield, and harvest index [81, 83]. In Group A, variety XM26, only containing *Rht4*, showed significantly higher MSSGN and TKW, compared to ZM155, SN086, and JM23, these varieties did not have *Rht4.* These suggest that future utilization of dwarfing genes should employ gene synergy or complementary strategies to mitigate the negative effects of individual dwarfing genes when reducing plant height of wheat, and maximize the optimization of plant architecture and grain yield.

## Conclusion

This study systematically analyzed 10 cloned *Rht* genes to elucidate their roles on molecule properties through comparative genomics and expression profiling, evaluate allelic effect of *Rhts* by association analysis of trait-alleles. Results reveal that *Rht12* and *Rht24* effectively mitigate the negative yield effects of *Rht1* or *Rht2*, although *Rht12* and *Rht24* exhibit relatively weak stem-shortening abilities. The combination of *Rht4* and *Rht8* optimizes plant architecture and enhances yield potential, while the triple-gene stack of *Rht8*, *Rht12* and *Rht24* achieves higher yield. Based on above results, prioritizing the aggregation of *Rht8*, *Rht12* and *Rht24* genotypes can achieve synergistic improvement of high yield and dwarf traits, significantly enhancing yield potential.

### Shortcomings and prospects

The molecular regulatory mechanisms of specific genes identified in this study require further elucidation. Although with the closely linked molecular markers, some genes may have a relatively low probability of genetic recombination, which may lead to erroneous judgement of genotyping results. Integrating diagnostic markers, haplotype analysis, and high-throughput sequencing technologies can systematically validate current findings. Based on the above results, in the breeding practices, efficient KASP molecular markers of key *Rht* genes should be exploited to select superior haplotypes, and genome editing technologies like CRISPR/Cas9 can be employed for precise editing of target *Rht* genes associated with plant height and grain yield, to produce superior genotypes. Future study should delve into the interaction mechanisms between *Rht* alleles and stress tolerance traits, such as pest and disease resistance, adverse environment adaptation, to elucidate the genetic basis for multi-trait synergistic improvement.

## Supplementary Information

Supplementary Material 1.

Supplementary Material 2.

Supplementary Material 3.

Supplementary Material 4.

Supplementary Material 5.

Supplementary Material 6.

Supplementary Material 7.

Supplementary Material 8.

Supplementary Material 9.

Supplementary Material 10.

Supplementary Material 11.

Supplementary Material 12.

## Data Availability

All data generated or analysed during this study are included in this manuscript and its supplementary information files.
