# Supplementary figures and images for: Molecular characteristics and regulatory effects of dwarfing Rht genes in Triticum aestivum L
Source: BMC Plant Biol. 2025 Nov 10;25:1529. doi: 10.1186/s12870-025-07556-w (PMC12599085; doi:10.1186/s12870-025-07556-w)

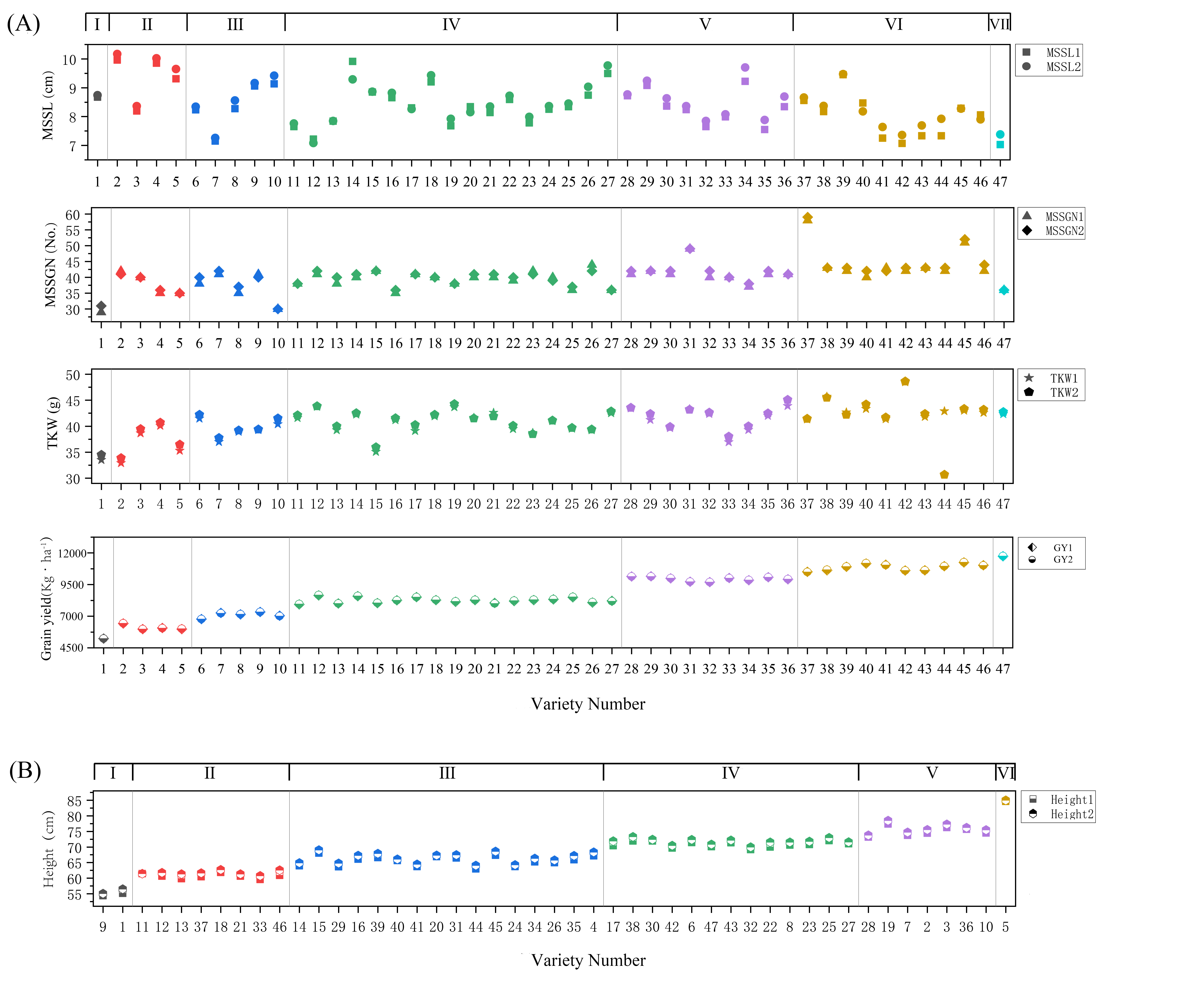

Supplement: Supplementary file 6 — Supplementary Material 6. [file 12870_2025_7556_MOESM6_ESM.jpg]
